# Isolation and Characterization of Lactic Acid Bacteria With Probiotic Attributes From Different Parts of the Gastrointestinal Tract of Free-living Wild Boars in Hungary

**DOI:** 10.1007/s12602-023-10113-2

**Published:** 2023-06-23

**Authors:** Tibor Keresztény, Balázs Libisch, Stephanya Corral Orbe, Tibor Nagy, Zoltán Kerényi, Róbert Kocsis, Katalin Posta, Péter P. Papp, Ferenc Olasz

**Affiliations:** 1 Agribiotechnology and Precision Breeding for Food Security National Laboratory, Institute of Genetics and Biotechnology, Hungarian University of Agriculture and Life Sciences (MATE), 2100 Gödöllő, Hungary; 2 Doctoral School of Biological Sciences, Hungarian University of Agriculture and Life Sciences, 2100 Gödöllő, Hungary; 3 Institute of Genetics and Biotechnology, Hungarian University of Agriculture and Life, Sciences, 2100 Gödöllő, Hungary; 4 Hungarian Dairy Research Institute Ltd, 9200 Mosonmagyaróvár, Hungary

**Keywords:** Lactic acid bacteria, Probiotics, Wild boar, Antimicrobial activity, pH and bile tolerance, Bile salt hydrolase (BSH) activity

## Abstract

Lactic acid bacteria (LAB) in the microbiota play an important role in human and animal health and, when used as probiotics, can contribute to an increased growth performance in livestock management. Animals living in their native habitat can serve as natural sources of microorganisms, so isolation of LAB strains from wild boars could provide the opportunity to develop effective probiotics to improve production in swine industry. In this study, the probiotic potential of 56 LAB isolates, originated from the ileum, colon, caecum and faeces of 5 wild boars, were assessed in vitro in details. Their taxonomic identity at species level and their antibacterial activity against four representative strains of potentially pathogenic bacteria were determined. The ability to tolerate low pH and bile salt, antibiotic susceptibility, bile salt hydrolase activity and lack of hemolysis were tested. Draft genome sequences of ten *Limosilactobacillus mucosae* and three *Leuconostoc suionicum* strains were determined. Bioinformatic analysis excluded the presence of any known acquired antibiotic resistance genes. Three genes, encoding mesentericin B105 and two different bacteriocin-IIc class proteins, as well as two genes with possible involvement in mesentericin secretion (*mesE*) and transport (*mesD*) were identified in two *L. suionicum* strains. Lam29 protein, a component of an ABC transporter with proved function as mucin- and epithelial cell-adhesion factor, and a bile salt hydrolase gene were found in all ten *L. mucosae* genomes. Comprehensive reconsideration of all data helps to select candidate strains to assess their probiotic potential further in animal experiments.

**Supplementary Information:**

The online version contains supplementary material available at 10.1007/s12602-023-10113-2.

## Introduction

A balanced and highly diverse microbiota is essential for healthy life of living organisms in the animal kingdom. Perturbed balance of the microbiota may lead to disease development. Conditions in industrial scale livestock production frequently result in increased stress and health problems. Farming conditions, environmental and nutritional effects and weaning difficulties are the main stress factors in the pig production sector affecting significantly animal health and production. The usage of antibiotics, to overcome stress related negative health effects as well as for growth promotion particularly, was banned in the European Union and in many other countries [1]. Probiotics, used as feed supplements, can serve as alternatives to antibiotics in various cases to avoid economic losses in swine production [2]. Probiotics have beneficial impacts on the balance of gastrointestinal microbiota, the ability to fight enteric pathogens, and capacity to support the immune system [3].

Lactic acid bacteria (LAB) are the most commonly used microorganisms as probiotics. LAB display a strong antimicrobial activity against a range of pathogenic microorganisms and can provide nutritional and health benefits to the hosts [4]. Among LAB, the most commonly used probiotic strains belong to lactobacilli [5], at a large extent due to the “Generally Regarded as Safe” (GRAS) status of these organisms [6]. Probiotic strains for pigs can improve growth performance, feed conversion efficiency, nutrient utilization, community structure of intestinal microbiota, gut health and regulate the immune system. There are several requirements for probiotic bacteria, the most important being that they must be harmless to the host, i.e. they must not have hemolytic activity or carry acquired antibiotic resistance determinants. It must also possess beneficial properties, such as antibacterial activity, pH and bile acid tolerance, vitamin production, immune-stimulatory activity. However, a given strain does need to possess all the beneficial properties. The roles of *Lactobacillus* sp. as probiotics for swine were reviewed widely [2, 7].

The source of probiotic strains is important, and they should be isolated from the intestinal microflora of the host animal species in order to achieve easier intestinal colonization and more specific applications. Host specificity was observed among lactobacilli isolated from human and animal sources [8]. Lactobacilli and streptococci isolated from swine have shown better adherence to swine squamous epithelial cells than strains isolated from other animals [9], indicating that survival of probiotic bacteria is dependent on a host-specific environment.

Although there is an extensive literature on the intestinal flora of domestic pig, the knowledge on the intestinal flora of wild boar, which are taxonomically the same species, is scarce. One of the main differences between domestic pigs and wild boars, apart from genetic differences, is that wild boars have to survive under natural conditions without the use of any feed additives, antibiotics or probiotics used in livestock production. Their microbiota may contain beneficial microorganisms that help to prevent various diseases and provide resistance to pathogens. As a result of domestication and prolonged selection in large-scale farming, the composition of the gut microbiota of domestic pigs has changed significantly, and bacterial strains with physiologically beneficial effects that are still found in natural populations may have been lost. Wild boars have a very diverse fecal bacterial community, probably due to their complex food structure [10], therefore it is possible to isolate microorganisms with different properties. Furthermore, wild boars have generally shown high tolerance and/or resistance to many diseases, suggesting that their gut bacteria may be a good source for isolation of potential probiotic strains. Therefore, we have chosen the intestinal contents of the different segments of the intestinal tract of wild boars as a source for the isolation of useful microorganisms. Recognition of the value of wild boar as a potential source of probiotic strains has been reported recently [11, 12].

The aim of the present study was to identify LAB isolated from wild boars and to screen them for probiotic attributes and safety properties in vitro*,* in order to select strains that can be used as direct fed microbials in the swine industry. The sampled wild boars were living in their native habitat, in one of the less human-affected regions in Hungary. Guidelines for the evaluation of probiotic activity of candidate strains, recommended by the Food and Agriculture Organization of the United Nations (FAO) [13] and reviewed in several articles [14–16], were followed to characterize the isolates. Determination of draft genome sequences and their bioinformatic analyses also facilitated the selection of candidate strains for further evaluation in animal experiments as direct-fed microbials for pigs.

## Materials and Methods

### Sources and Collection of Samples

Samples were taken from 5 (#1 - #5) wild boars (*Sus scrofa*) living in a native habitat of the Zemplén Mountain in Hungary near to the Slovakian border [17]. Wild boars were shot during regular hunts organized by the local hunting party in Füzérkomlós, the rightful owner of this hunting area for hunting with reference to the LV of 1996 Hungarian State Law on wildlife protection, wildlife management, and hunting, where the boars were professionally eviscerated. All the animals were healthy except for boar #1, which showed signs of enteritis with diarrhea [17]. Four of the wild boars were sows (#1 - #3 and #5) and one boar was male (#4). Three of the sampled animals were adults (#1 - #3), while two (#4 and #5) were young animals. During sampling, the parts of the intestinal tract designated for sampling were sealed with forceps, excised and transported to the laboratory under refrigerated and anaerobic conditions using anaerobic jars and processed within 24 h. The intestinal contents from ileum, colon, caecum and rectum were removed under sterile conditions.

### Isolation and Identification of Lactic Acid Bacteria

#### Handling and Storage of Bacterial Samples

One gram from each sample was aseptically added to 5 mL of sterile phosphate buffered saline (PBS) solution (pH 7.2) and mixed thoroughly. Serial dilutions were performed and 0.1 mL aliquots from the dilutions were plated out onto different kinds of media such as BHI – Brain Heart Infusion Agar (Biolab Ltd., Hungary), TSA – Tryptic Soy Agar (Biolab Ltd., Hungary), M17 Agar (Biolab Ltd., Hungary), MRS – De Man, Rogosa and Sharpe Agar (BIOKAR Diagnostics, France), MRSCC – MRS agar supplemented with 5-5 g L^−1^ maltose, fructose, sucrose and 0.05 g L^−1^ cysteine-HCL (Sigma-Aldrich, Japan) and EA – Elliker Agar (Biolab Ltd., Hungary) in triplicate by spreading method. One set of the plates was incubated in an aerobic thermostat, the second set was placed into a CO_2_ (5 %) incubator (Sheldon Manufacturing, Inc., OR, US) (semi-anaerobic condition) and the third set of plates was placed into anaerobic jars supplemented with AnaeroGen 2.5 L sachets (Oxoid, Basingstoke, UK). Incubation was carried out at 35 °C for 48-72 hrs.

Single colonies (without selection) were suspended in 105 μL PBS buffer, then 45 μL of 50 % glycerol was added and the tubes were placed into a -70 °C freezer for storage.

#### Screening and Species-level Determination of Isolates By 16S Sanger Sequencing

Polymerase chain reaction (PCR) was used for determination of the 16S sequence of the isolates. A single colony was suspended in 100 μL peptone saline water (0.85 % NaCl and 0.1 % peptone) buffer. PCR was carried out using DreamTaq 2X PCR master mix (Thermo Scientific Ltd, UK) according to manufacturer’s instructions. As template, 1 μL of cell suspension from each strain in the collection was used in 20 μL reaction. Primer Lac1F (5’-AGCAGTAGGGAATCTTCCA-3’) and Lac2GCR (5’-ATTYCACCGCTACACATG-3’) [18] were used for screening LAB strains, Leucgrp fw (5’-GCGGCTGCGGCGTCACCTAG-3’) and Leucgrp rev (5’-GGNTACCTTGTTACGACTTC-3’) primers [19] were used to identify isolates belonging to the *Leuconostoc* genus. Universal primer pair, 27f (5’-AGAGTTTGATCCTGGCTCAG-3’) and 1492r (5’-GGTTACCTTGTTACGACTT-3’) [20] was used for amplification of the 16S rDNA. PCR conditions were the same as given in the papers reporting the primer pairs. The PCR products were purified using MinElute PCR Purification Kit (QIAGEN, Germany) according to the manufacturer’s instructions, and then sequenced with both primers (27f and 1492r). Sequencing of the amplicons was performed using Big-Dye Sequencer ABI 3130xl by BIOMI Ltd. (Gödöllő, Hungary). Sequence homologies were examined by comparing the sequences obtained with those of the NCBI databases (Reference RNA Sequences (refseq_ma) and RefSeq Genome Database (refseq_genomes)) using Basic Local Alignment Search Tool (BLAST) software [21] and identified according to the closest relative.

### Screening of Isolates for Probiotic Attributes

#### Demonstration of Antimicrobial Activities

To assess the antimicrobial activity of the isolates the agar well diffusion assay [22] was applied. The isolates were inoculated to MRSCC broth at 35 °C for 24 h and the cell-free supernatants were collected by centrifugation (15,600×g, 10 min) to use in the test. Out of the 4 tester strains, *Escherichia coli* W3110 [23]*, Staphylococcus aureus* SP17 [24] and *Salmonella enterica* serov. Typhimurium LT2 (ATCC 700720) were cultured in Luria-Bertani (LB) broth [25] and plated on LB agar, while M17 media (broth and agar) (Biolab Ltd., Hungary) were used to work with *Streptococcus thermophilus* T9 (Hungarian Dairy Research Institute Ltd, Hungary). After spreading 0.1 mL of overnight grown cultures from the tester strains on the agar plates by soft agar overlay, wells of 5 mm diameters were punched. Aliquots (80 μL) from the supernatants of the LAB cultures were dispensed into the wells, and the plates were incubated for 24 h at 35 °C and the appearance of a clearing zone was examined. The diameter of clear inhibition zone around the wells was measured with a ruler and isolates were rated as –, +, ++ and +++ (–: 6 mm; +: >6-9 mm; ++: 9-15 mm and +++: >15 mm).

#### Determination of Acid Tolerance of Selected Isolates

The acid tolerance of the isolates was tested at pH 2.0 and 3.0 in pilot experiments and finally measured accurately at pH 2.5. The isolates were sub-cultured in MRSCC broth at 35 °C for 24 h. From the cultures, 60 μL were added to 3 mL of MRSCC broth which was adjusted to pH 2.5. Viable count was conducted by counting colony forming units (CFU) on MRSCC agar plates from 20 μL aliquots taken immediately after inoculation and after 1 and 2 hours of incubation. Liquid cultures were incubated in CO_2_ incubator while plates were incubated in anaerobic jars (Oxoid, UK) containing AnaeroGen™ 2.5 L sachet (Thermo Scientific, UK).

#### Bile Tolerance Assay to Determine the Sensitivity of Isolates

To evaluate the ability of isolates growing in the presence of bile salt, isolates were cultured in MRSCC broth supplemented with 0.3 and 1.0 % (w/v) bile salt (bovine, Sigma-Aldrich, USA). Viable count was conducted by counting colony forming units on MRSCC agar plates from 20 μL aliquots taken after 4 and 7 hours of incubation. Liquid cultures were incubated in CO_2_ incubator while plates were incubated in anaerobic jars (Oxoid, UK) containing AnaeroGen™ 2.5 L sachet (Thermo Scientific, UK).

### Tests for Addressing Safety Concerns

#### Antibiotic Susceptibility Testing to Detect Certain Acquired Antibiotic Resistances

The selection of antibiotics was based on previous publications [26–28]. These publications were designed to detect antibiotics specific to Gram-positive bacteria and representing multiple antibiotic types. Susceptibility of strains to selected antibiotics was evaluated by soft agar overlay disc diffusion method on MRSCC agar [26], with slight modifications (Petri plates, containing 20 mL agar, were overlaid with 3 ml of 0.8 % soft agar seeded with 50 μL of an active culture at 45 ℃). The following antibiotic discs (BIO-RAD, France) were used: erythromycin (15 μg), kanamycin (30 μg), tetracycline (30 μg), trimethoprim (5 μg), vancomycin (30 μg), oxacillin (5 μg) and azithromycin (15 μg). The diameters of inhibition zones were determined after anaerobic incubation at 35 ℃ for 24 h by using a Scan 500 inhibition zone reader (Interscience, France). Strains were categorized as resistant (R), intermediate (I) or susceptible (S) according to interpretative standards described previously [27]. The interpretation for oxacillin and azithromycin were based on data reported by the National Committee for Clinical Laboratory Standards [29] for staphylococci, as no other data of inhibition zone diameters for *Limosilactobacillus* were found in the literature. However, LAB strains showed various patterns when disks of 1 μg oxacillin were used [30, 31]. Quality was controlled by using *Staphylococcus aureus* ATCC 25923 for oxacillin and azithromycin and *Enterococcus faecalis* ATCC 19433 for the rest.

#### Monitoring the Presence of Bile Salt Hydrolase (BSH) Activity

BSH activity of candidate probiotics can be evaluated by the well-established agar plate assay [32]. In the setup of the semi-qualitative assay, we followed the report of [33] with some modifications. Sterile filter paper discs of 8 mm diameter were impregnated with 20 μL of cultured isolates on MRSCC agar (supplemented with 0.5 % sodium salt of taurodeoxycholic acid (Sigma-Aldrich, MO, USA) and 0.37 % CaCl_2_) plates. Plates were incubated at 35 °C for 72 h in anaerobic condition and the diameters of the precipitation zones around the disks were measured and rated as –, +, ++ and +++ (–: ≤ 14 mm, no precipitation zone; +: 14-24 mm; ++: 24-29 mm and +++: > 29 mm). Results were obtained and averaged from three independently repeated experiments.

#### Blood Hemolysis Test to Confirm the Safe Use of Isolates

Isolates were cultured in MRSCC broth for 24 h at 35 °C and the streak plate methods were performed on Columbia agar (Biolab Ltd., Hungary) plates supplemented with sterile sheep blood (5 % v/v) and kept for anaerobic incubation at 35 °C for 48 h. Hemolytic activity was judged by visual observation using *Salmonella enterica* serov. Typhimurium LT2 and *Staphylococcus aureus* SP17 strains as positive controls, showing complete hemolysis.

### Genome Sequencing and Bioinformatics Analyses

#### Sequence Determination of Selected LAB Strains

The complete genome sequence of ten *Limosilactobacillus mucosae* (formerly L*actobacillus mucosae*) [34] and three *Leuconostoc suionicum* (formerly *Leuconostoc mesenteroides* subs*. suionicum*) [35] strains were determined. Strains were grown using MRSCC broth medium at 35 °C for 24 h, then the cells were harvested and kept frozen in a –70 °C freezer until delivered to the sequencing facility. DNA preparation and sequence determination of the strains were performed by Xenovea Ltd. (Szeged, Hungary), except strain F1 which was performed by BIOMI Ltd. (Gödöllő, Hungary). De novo assembly of the raw reads was performed with SPAdes v3.0.0 software [36, 37] (https://cab.spbu.ru/software/spades/). After the *de novo* assembly, contigs below 500 bp were filtered out by using in-house Linux scripts. Contamination was removed using a BLAST search against the eukaryotic RefSeq sequences. RefSeq sequences were downloaded from NCBI. If the alignment length of the match was bigger than the half of the contig’s length we marked the scaffold as contamination and removed it from further analysis. The sequences of the cleaned draft genomes were deposited in the NCBI database, under accession number PRJNA926800.

#### Bioinformatics Analyses of Sequenced Potential LAB Strains

##### K-Mer Analyses

Draft genome sequences of the strains were analyzed by KmerFinder 3.2 program (Software version 3.0.2) [38–40]. The service was available at the homepage of Center for Genomic Epidemiology (https://cge.food.dtu.dk/services/KmerFinder/). Sequences in FASTA format were uploaded and compared to sequences in the “Bacteria organism” database (version 2022-07-11). “Query Coverage (%), the percentage of input query Kmers that match the template” in the output of the results, was used to evaluate taxonomical relationships at strain level.

##### Phylogenetic Analyses

Whole genome alignment was performed using progressiveMauve [41] (build date: Feb 13 2015). The command lines were the following for *Limosilactobacillus*:

progressiveMauve --output-guide-tree=Limosilactobacillus.tree --output Limosilactobacillus *.fasta

and for *Leuconostoc*:

progressiveMauve --output-guide-tree=Leuconostoc.tree --output Leuconostoc *.fasta

The guided tree was used to create trees which were drawn by using the ape package from R [42]. The used commands were the following for *Limosilactobacillus*:tree1 <- read.tree(“Limosilactobacillus.tree”); plot(tree1)and for *Leuconostoc*:

tree2 <- read.tree(“Leuconostoc.tree”); plot(tree2)

Similarity matrixes were created by using FastANI (version 1.33) [43]. The program calculates the average nucleotide identity for every sequence pair. The query and reference dataset were the same, they contained sequences of selected publicly available strains and of our sequenced strains. The command line options were the following for *Limosilactobacillus*:

fastANI --ql Limosilactobacillus.txt --rl Limosilactobacillus.txt -o Limosilactobacillus.out --matrix -t 10

and for *Leuconostoc*:

fastANI --ql Leuconostoc.txt --rl Leuconostoc.txt -o Leuconostoc.out --matrix -t 10

##### **Screening for the Presence of Antibiotic Resistance Genes**

Draft genome sequences of the strains were analyzed by ResFinder 4.1 software [44–46] to uncover the presence of any acquired antibiotic resistance gene. The service, available at the homepage of “Center for Genomic Epidemiology” (https://cge.food.dtu.dk/services/ResFinder/), was used with the following settings: selected %ID threshold 90 %, selected minimum length 60 %. ResFinder database: (2022-05-24). Sequences in FASTA format were uploaded according to instructions of the service provider.

##### Screening for Known Bacteriocin Genes

BAGEL4 web server (http://bagel4.molgenrug.nl/) has the ability for searching bacteriocins and RiPPs (ribosomally synthesized and posttranslationally modified peptides) [47]. Genome sequences were uploaded in FASTA format. Analyses were focused on genes with possible involvement in biosynthesis and functioning of bacteriocins.

##### **Genes with Potential Relevance for Probiotics**

A 29-kDa protein (Lam29) from *L. mucosae* ME-340 is an ABC transporter component, but it has also been shown to function as mucin- and epithelial cell-adhesion factor [48, 49]. Sequence of the *lam29* gene was identified in each *L. mucosae* draft genome sequence by BLAST search. Genes were cut using seqret program [50]. Multiple alignment was constructed using Mafft (version 7.429) [51] with the following parameters:

--treeout --thread 10 --localpair --maxiterate 2000 --clustalout –reorder

The final tree was drawn using the ape package of R [42].

BSH genes were identified using Tblastn [52] search, because the query sequence was protein. Seqret from Emboss [50] was used to cut the region of the genes. Multiple alignment was constructed using Mafft with the same parameters as mentioned above. The final tree was drawn using the ape package of R [42].

#### Data Availability of Draft Genome Sequences

Tha draft genome sequences are available at NCBI under the BioProject: PRJNA926800.

## Results

### Isolation and Identification of Lactic Acid Bacteria

#### Bacterial Isolates and Their Screening By PCR Amplification

A primary strain collection of 4334 isolates has been established from colonies of different kinds of bacteria, originated from four locations of the gastrointestinal tracts (ileum, colon, caecum and rectum) of five wild boars living in natural habitat. Altogether, 1650 ileum, 1049 colon, 727 caecum and 908 rectum isolates were collected; 1128, 1624, 655, 409 and 518 derived from wild boar #1 to wild boar #5, respectively. Aerobic condition provided 2738 isolates, while semi-anaerobic and anaerobic gave 436 and 1160 ones, respectively.

For the screening of isolates from the strain collection, isolates belonging to four genera such as *Lactobacillus*, *Pediococcus*, *Leuconostoc* and *Weissella* could be identified by LAB specific PCR*.* PCR positive isolates were purified by sub-culturing and plating until pure colonies were obtained, and a secondary strain collection (894 isolates) has been created from pure cultures. For further screening and characterization, 166 strains were selected (Supplementary Table 1). PCR specific for *Leuconostoc* genus was suitable to identify 20 strains which belong to this genus (Supplementary Table 1), while classification of the rest of the isolates required other sequence-based method, such as 16S sequencing.

#### Demonstration of Antimicrobial Activities of the Selected Isolates

Ability of the 166 LAB strains to produce any antimicrobial compound was tested against 4 tester strains, *Escherichia coli* W3110*, Staphylococcus aureus* SP17, *Salmonella enterica* serov. Typhimurium LT2 (ATCC 700720) and *Streptococcus thermophilus* T9. The antimicrobial activity was quantified by measuring the diameter of the inhibition zone and rated as -, +, ++ and +++.

Of the 166 strains, 164 exerted antimicrobial activity against *Escherichia coli* and 158 against *Salmonella enterica* and *Staphylococcus aureus*. It was found that 64 strains had inhibitory effect on the growth of *Streptococcus thermophilus* as well (Supplementary Table 1).

The 166 isolates were grouped according to their antibiotic activity patterns. The 20 *Leuconostoc* isolates were classified into 3 groups while the other LAB isolates were classified into 46 groups (results are presented in Table 1 and Supplementary Table 1).

**Table 1:** Grouping of 56 selected LAB isolates based on pattern of their antimicrobial activity using four tester strains

**Strain**^***a***^		**Antimicrobial activity**^***c***^** against**			
ID	Origin^*b*^	*Escherichia coli*	*Salmonella enterica*	*Staphylococcus aureus*	*Streptococcus thermophilus*
F1	caecum	**+++**	**+++**	**+++**	**+++**
F2 F4 F6	ileum ileum ileum	**+++** **+++** **+++**	**++** **++** **++**	**+++** **+++** **+++**	**+++** **+++** **+++**
F7 F9 F10 F13 F14	rectum caecum caecum ileum ileum	**+++** **+++** **+++** **+++** **+++**	**+++** **+++** **+++** **+++** **+++**	**++** **++** **++** **++** **++**	**+++** **+++** **+++** **+++** **+++**
F15	ileum	**+++**	**+**	**+++**	**+++**
F16	ileum	**+++**	**+++**	**+**	**+++**
F17	caecum	**+++**	**+++**	**+++**	**-**
F18 F20	caecum ileum	**+++** **+++**	**+++** **+++**	**++** **++**	**++** **++**
F23 F24	caecum ileum	**+++** **+++**	**++** **++**	**++** **++**	**+++** **+++**
F29	ileum	**+++**	**++**	**+++**	**+**
F31	ileum	**+++**	**+**	**+++**	**++**
F35	caecum	**+++**	**++**	**+**	**+++**
F45 F48 F49	colon ileum ileum	**+++** **+++** **+++**	**+++** **+++** **+++**	**++** **++** **++**	**-** **-** **-**
F52	ileum	**+++**	**++**	**+++**	**-**
F61	ileum	**+++**	**+**	**+++**	**-**
F65	ileum	**+++**	**++**	**++**	**++**
F66	caecum	**++**	**++**	**++**	**+++**
F68	ileum	**+++**	**++**	**++**	**+**
F69	ileum	**+++**	**+**	**++**	**+**
F71	colon	**++**	**+**	**+**	**+++**
F79	rectum	**+**	**+**	**+**	**+++**
F84 F88 F98 F105	colon colon ileum ileum	**+++** **+++** **+++** **+++**	**++** **++** **++** **++**	**++** **++** **++** **++**	**-** **-** **-** **-**
F108	ileum	**++**	**+++**	**++**	**+**
F113	ileum	**++**	**+**	**+++**	**-**
F116	caecum	**++**	**++**	**++**	**++**
F120	colon	**++**	**+**	**+**	**++**
F122	colon	**+**	**+**	**++**	**+**
F126 F132	caecum ileum	**++** **++**	**++** **++**	**++** **++**	**-** **-**
F133	colon	**++**	**++**	**+**	**-**
F137	ileum	**++**	**++**	**-**	**-**
F138	ileum	**++**	**-**	**-**	**-**
F139	rectum	**+**	**+**	**+**	**+**
F144	caecum	**+**	**-**	**-**	**-**
F146	caecum	**-**	**-**	**-**	**-**
F147 F148 F150	caecum caecum caecum	**+++** **+++** **+++**	**++** **++** **++**	**++** **++** **++**	**-** **-** **-**
F151	rectum	**+++**	**++**	**+**	**-**
F156 F158 F162 F163 F166	caecum rectum rectum rectum rectum	**++** **++** **++** **++** **++**	**+** **+** **+** **+** **+**	**+** **+** **+** **+** **+**	**-** **-** **-** **-** **-**

^*a*^taxonomic identification of the strains is based on 16S rDNA sequencing. F1-F146 are *Limosilactobacillus mucosae*, F147-F166 are *Leuconostoc suionicum* strains

^*b*^gut region of sample origin

^*c*^activity is rated according to the size of clear inhibition zone around the wells, diameter rating is: –: 6 mm; +: >6-9 mm; ++: >9-15 mm and +++: >15 mm

There were 56 isolates (9 *Leuconostoc* and 47 other LAB strains) selected for further characterization which represented the majority of the groups (Table 1). In addition to the obtained rating patterns of antimicrobial activity, origin of the samples was also taken into consideration to cover as many gut regions as possible from each wild boar.

#### Taxonomic Identification of Selected Isolates

Sequencing of the 16S rRNA gene region (~1380±40 bp sequence between nucleotides 27 and 1492) from each selected strain was used for taxonomic identification at species level. Comparison of the obtained sequences to sequences in NCBI databases resulted in the identification of 47 *Limosilactobacillus mucosae* (*L. mucosae*) and 9 *Leuconostoc suionicum* (*L. suionicum*) strains (Table 1 and Supplementary Table 1). BLAST search in both refseq_ma and refseq_genomes databases gave concordant results for *L. mucosae* strains.

In the case of *Leuconostoc* strains, searches in the refseq_ma database identified the strains as a member of *Leuconostoc mesenteroides* species with 99.93 % identity, while searches in the refseq_genomes database identified the strains as a member of *L. suionicum* species with 100 % identity. We have accepted *L. suionicum* species as the closest relatives.

### Screening of Isolates for Probiotic Attributes

#### Determination of Acid Tolerance of the Isolates

The effect of acidity on the viability of the selected isolates was assessed by measuring their growth at pH 2.5 in MRSCC broth. Viable colony forming units were detected in aliquots collected immediately after inoculation and after 1 and 2 hours of incubation, reflecting the time spent by food in the stomach [53]. The experiment was also performed at pH 2.0 and pH 3.0, but the results were inconclusive. Essentially, at pH 3.0 there were no significant difference between viability of the isolates, whereas at pH 2.0 all strains were seriously affected after 1 h (data not shown).

The majority of *L. mucosae* strains tolerated pH 2.5 for 2 hours very well, as viable counts were close to or even higher than the starting state (over 0.7 log unit increase in the case of F4, F23, F49, F68, F116 and F132), while in some cases (F10, F105, F120 and F122) a significant reduction (1-3 log units) was observed (Supplementary Table 2).

*L. suionicum* strains were rather sensitive to low pH exposure, as differences were visible after 1 h incubation. The viability of four strains was reduced with 1.5-2.1 log units (F150, F151, F156 and F163) while the other five strains lost their viability entirely. Further incubation eliminated viability of all strains (Supplementary Table 2).

#### Identification of Isolates Tolerant to Bile Stress

When the experimental parameters were determined, the bile salt concentration of 0.3 % was considered as physiological condition in mammalian guts [54], whereas 1.0 % bile salt concentration represented a high stress condition. The 7 h incubation time resemble to the time that bacteria spend in the gut region where high bile salt stress condition occurs.

At 0.3 % bile salt concentration, 28 out of the 56 strains showed increased viable counts after 7 h incubation compared to initial counts, two of them over 0.8 log units (F139 and F151) (Table 3). Decrease in viable counts were less than 0.2 log unit in the case of another 12 strains. Only 3 strains (F13, F45 and F69) suffered over 1.0 log unit decrease in viable counts at this condition (Supplementary Table 3).

At 1.0 % bile salt concentration, only three *L. mucosae* (F20, F84 and F88) and two *L. suionicum* (F151 and F158) strains were able to maintain or exceed initial counts after 7 h incubation. Six *L. mucosae* (F2, F4, F7, F35, F45 and F133) and two *L. suionicum* (F150 and F156) strains showed less than 0.4 log unit decrease. These 13 strains were considered to be highly tolerant against bile stress. Four *L. mucosae* strains (F15, F16, F71 and F132) were rather sensitive against bile stress (over 2.5 log units decrease in viable counts) (Supplementary Table 3).

### Tests for Addressing Safety Concerns of LAB Strains

#### Antibiotic Susceptibility Testing to Determine Resistance Pattern of Isolates

**Fig. 1:**
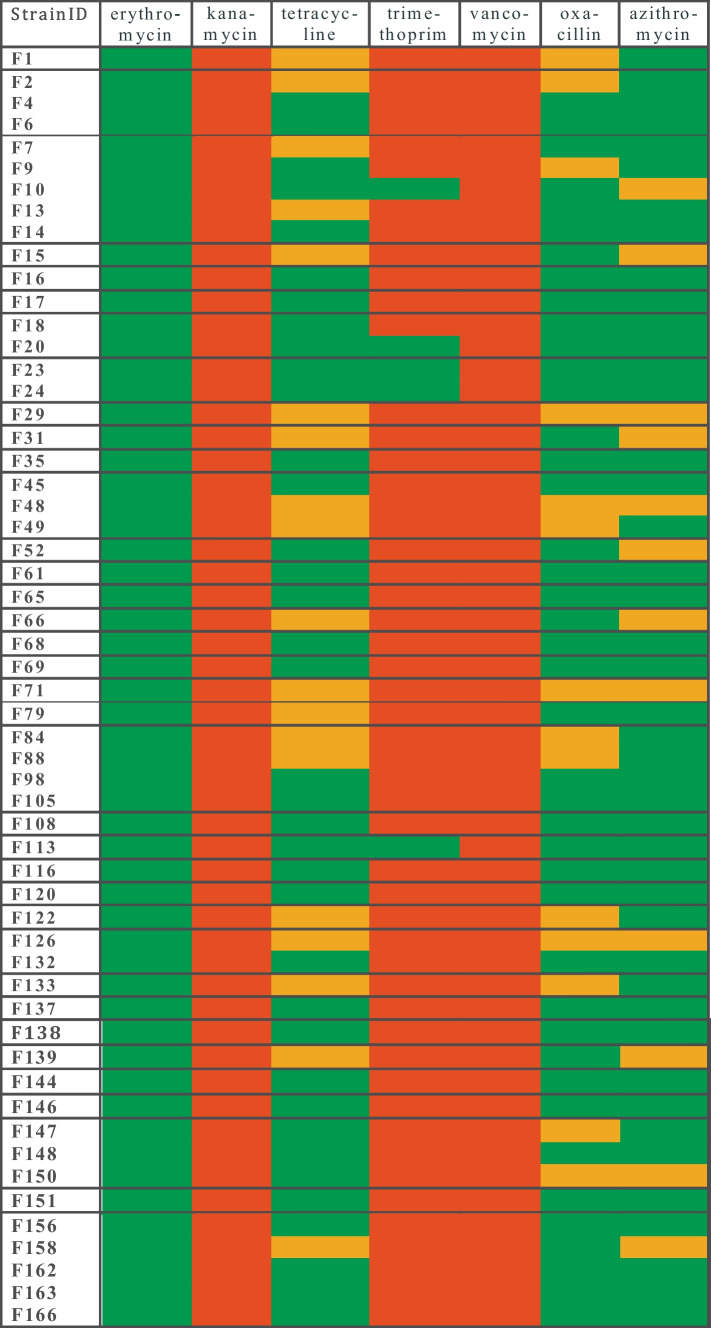
Heat map of antibiotic susceptibility classification of selected isolates. F1-F146 are *L. mucosae*, while F147-F166 are *L. suionicum* strains. Horizontal lines maintain and separate groups established according to antimicrobial patterns of the strains. Strains in a group have identical antimicrobial pattern for the four tester strains used. (Table 1 and Supplementary Table 1). Color coding: red = resistant; orange = intermediate and green = susceptible

We have tested the 56 strains for 7 antibiotics (erythromycin, kanamycin, tetracycline, trimethoprim, vancomycin, oxacillin and azithromycin) by disc diffusion method and the results are shown in Fig.1. All strains were resistant to vancomycin and kanamycin, however, we have found 5 *L. mucosae* strains (F10, F20, F23, F24 and F113) sensitive to trimethoprim, while the rest of the strains were resistant. All strains were sensitive to erythromycin. There were no strains resistant to tetracycline, oxacillin and azithromycin but the phenotype distribution of *L. mucosae* strains was 29 sensitive and 18 intermediate to tetracycline, 35 sensitive and 12 intermediate to oxacillin and 37 sensitive and 10 intermediate to azithromycin, according to criteria published in [27]. *L. suionicum* strains were mainly sensitive to these three antibiotics except for 1 intermediate to tetracycline (F158) and 2-2 intermediate to oxacillin (F147 and F150) and azithromycin (F150 and F158).

#### Monitoring the Presence of Bile Salt Hydrolase (BSH) and Blood Hemolysis Activity

Out of the 56 isolates tested for BSH activity, none of the 9 *L. suionicum* strains were positive, while all the 47 *L. mucosae* strains expressed reasonable activity. Strains F14, F16, F18, F69, F79 and F105 showed below average, while F10, F35, F49, F133, F139 and F146 showed above average BSH activity (Table 2).

**Table 2:** BSH activity rating of selected *L. mucosae* (F1-F146) and *L. suionicum* (F147-F166) strains

strain ID	rating^*a*^	strain ID	rating^*a*^	strain ID	rating^*a*^	strain ID	rating^*a*^
F1	++	F23	++	F71	++	F137	++
F2	++	F24	++	F79	+**	F138	++
F4	++	F29	++	F84	++	F139*	+++
F6	++	F31	++	F88	++	F144	++
F7	++	F35*	+++	F98	++	F146	+++
F9	++	F45	++	F105**	+	F147	-
F10*	+++	F48	++	F108	++	F148	-
F13	++	F49*	+++	F113	++	F150	-
F14	+**	F52	++	F116	++	F151	-
F15	++	F61	++	F120	++	F156	-
F16	+**	F65	++	F122	++	F158	-
F17	++	F66	++	F126	++	F162	-
F18	+**	F68	++	F132	++	F163	-
F20	++	F69	+**	F133*	+++	F166	-

^*a*^rating of precipitation zones around the disks: –: ≤ 14 mm, no precipitation zone; +: 14-24 mm; ++: 24-29 mm and +++: > 29 mm

^*^strains with high BSH activity

^**^strains with low BSH activity

It is important that probiotic bacteria do not cause lyses of red blood cells in the host organism. Although there are no published reports on the hemolytic activity in lactobacilli so far, for safety reasons, the analyses cannot be avoided. None of the strains, except positive controls (*Salmonella enterica* serov. Typhimurium LT2 and *Staphylococcus aureus* SP17), showed hemolytic activity under the assay conditions.

### Genome Sequencing and Bioinformatics Analyses

#### Whole Genome Sequencing of Selected Isolates

Ten *L. mucosae* and three *L. suionicum* strains were selected for whole genome sequencing. Features of sequence assembly are shown in Table 3, and the sequences were deposited in NCBI database (accession number PRJNA926800). The relatively low contig numbers and the size of longest contigs indicate that sequencing quality meets the general standards. The genome size range of *L. mucosae* strains is 1.86-2.45 Mb [55], and the observed genome size of our strains fell in the range of 2.08-2.32 Mb (Table 3), while the genome size of *L. suionicum* falls between 2.02 and 2.13 Mb according to ten known strains available in NCBI database, our genomes have a size range of 2.13-2.16 Mb (Table 3).

**Table 3:** Features of sequence assembly of ten *L. mucosae* and three *L. suionicum* genomes after eliminating small (< 500 bp) and contaminant contigs

***Strain ID***	***Species***	***Number of contigs***	***Longest contig (bp)***	***Total length (bp)***	***Similarity (%) to*** ***L. mucosae LM1 by***	
					***K-mer***^***a***^	***FastANI***^***b***^
**F1**	*L. mucosae*	36	335 235	2 035 530	18.07	88.03
**F2**	*L. mucosae*	26	330 013	2 132 369	20.88	88.72
**F4**	*L. mucosae*	60	280 701	2 165 593	19.23	88.31
**F7**	*L. mucosae*	24	520 166	2 117 367	70.15	97.43
**F17**	*L. mucosae*	48	247 731	2 185 230	18.20	88.11
**F20**	*L. mucosae*	25	335 771	2 078 702	18.06	88.15
**F45**	*L. mucosae*	43	343 086	2 260 567	17.33	88.05
**F88**	*L. mucosae*	31	476 267	2 316 090	66.67	97.56
**F108**	*L. mucosae*	34	513 221	2 312 507	65.35	97.62
**F146**	*L. mucosae*	28	330 013	2 263 663	16.34	88.16
***Strain ID***	***Species***	***Number of contigs***	***Longest contig (bp)***	***Total length (bp)***	***Similarity (%) to L. suionicum* DSM 20241 by**	
					***K-mer***^***a***^	***FastANI***^***b***^
**F150**	*L. suionicum*	10	803 932	2 156 525	64.03	97.64
**F151**	*L. suionicum*	16	877 041	2 128 721	64.74	97.59
**F156**	*L. suionicum*	11	803 932	2 156 765	64.01	97.64

^a^ values of best match (similarity %), to strain *L. mucosae* LM1 (Accession No: NZ_CP011013.1) in the cases of *L. mucosae* species, and to strain *L. suionicum* DSM 20241 (Accession No: NZ_CP015247.1) in the cases of *L. suionicum* species, derived from K-mer analyses

^b^ similarity values were also obtained from the data matrix generated by FastANI during comparison of the entire genome sequences (Supplementary Table 4)

#### Taxonomic Analyses of Isolates Based On Whole Genome Sequencing

K-mer analysis can identify the closest relatives of a query sequence within a targeted database, in our case the “Bacteria database”. All ten *L. mucosae* strains showed the best match to *L. mucosae* LM1 strain (Accession No: NZ_CP011013.1), however, F7, F88 and F108 comprise a cluster with values within the 65-71 % range, while the other 7 strains formed a different cluster with values in the 16-21 % range (Table 3). All three *L. suionicum* strains showed the best match to *L. mesenteroides* subsp. *suionicum* DSM 20241 (Accession No: NZ_CP015247.1). Due to a recent update in the NCBI database, accession number NZ_CP015247.1 now represents the genome sequence of *L. suionicum* DSM 20241. The K-mer analyses confirmed the results of our previous, 16S sequence-based species identification as well as identified the closest known relatives in the database at strain level.

#### Phylogenetic Relationships of the Sequenced Strains

**Fig. 2:**
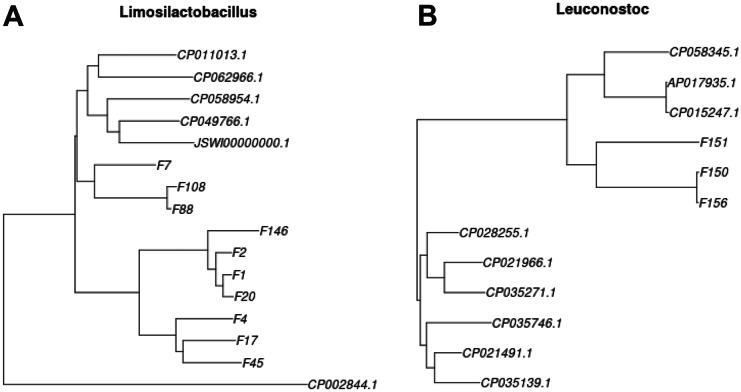
Phylogenetic relationship of *Limosilactobacillus* (**A**) and *Leuconostoc* (**B**) strains based on comparison of whole genome sequences. CP011013.1 indicates the position of *L. mucosae* LM1 (Accession No: NZ_CP011013.1) in part **A** and CP015247.1 indicates the position of *L. suionicum* DSM 20241 (Accession No: NZ_CP015247.1) in part **B**, which were the best matching strains according to the K-mer analyses. Strain sequences used for comparison with genome sequences of our strains were obtained from the NCBI database and marked with their accession numbers. *L. reuteri* SD2112 (CP002844.1) was used as outgroup control, and CP062966.1, CP058954.1, CP049766.1 and JSWI00000000.1 refer to *L. mucosae* strains, such as LM011, A1, L1 and DPC6426, respectively in part **A**. CP058345.1 refers to *Leuconostoc* sp. MTCC 10508, reclassified as *L. suionicum* [56], and AP017935.1 refers to *L. suionicum* LT-38, while CP028255.1, CP021966.1, CP035271.1, CP035746.1, CP021491.1 and CP035139.1 refer to *L. mesenteroides* strains, such as SRCM102735, CBA7131, SRCM103453, SRCM103460, WiKim33 and SRCM103456, respectively in part **B**

Sequence comparison of the draft genome of the strains by FastANI (version 1.33) program revealed their relationships. Similarity matrixes, based on the average nucleotide identity for each sequence pair, were obtained (Supplementary Table 4) and relationship between the strains was demonstrated by phylogenetic trees (Fig. 2A and B).

The *L. mucosae* strains F7, F88 and F108 form a subclade, which is clustered together with certain known *L. mucosae* strains available in the database such as LM1 (NZ_CP011013.1), LM011 (CP062966.1), L1 (CP049766.1), A1 (CP058954.1) and DPC6426 (JSWI00000000.1). The other 7 sequenced strains form another subclade but with higher diversity in their genome sequences (Fig. 2A). However, the subclade of 7 strains could be divided further into two clusters, one containing the genomes of the F1, F2, F20 and F146 strains while the other one the genomes of the F4, F17 and F45 strains. It is worth noting that clustering of the strains was completely independent from their origin within the gastrointestinal tract.

*L. suionicum* strains F150, F151 and F156 clustered together with *L. suionicum* DSM 20241 (NZ_CP015247.1) and other *L. suionicum* strains such as MTCC 10508 (CP058345.1) and LT-38 (AP017935.1) but they were completely distinct from the group of *L. mesenteroides* strains (Fig. 2B).

#### Testing for the Presence of Transmissible Antibiotic Resistance Genes

Draft genome sequences were analyzed to identify strains carrying potentially transmissible antibiotic resistance determinants. ResFinder 4.1 was applied and the results did not provide evidence for the presence of any known acquired antibiotic resistance gene in the strains investigated (data not shown). Consequently, our strains can be considered safe regarding possible transmissible antibiotic resistances.

#### Screening for Known Bacteriocin Genes

The BAGEL4 web tool was used to identify genes with possible bacteriocin function in the sequences of the strains. There were no hits with significant similarity (protein BLAST results were below 50 % match) to any known bacteriocin protein or peptide in the sequences of the *L. mucosae* strains. The annotation of the LM1 sequence, their closest relative *L. mucosae,* was also checked in the database and the result was also negative.

In the case of *L. suionicum* strains, no bacteriocin gene was detected in F151 according to BAGEL4 search, while F150 and F156 showed identical, positive results. Both strains have the same identical bacteriocin genes, encoding a 53 amino acid (AA) long peptide with 96.23 % identity to mesentericin B105 according to BAGEL4 and lactococcin family bacteriocin (Accession number: WP_032489578) according to protein BLAST. In the close proximity of this gene (1310 bp downstream), two tandem open reading frames were identified. The *mesD* gene, encoding a 722 AA long protein, showed 84.10 % identity to mesentericin Y105 transport/processing ATP-binding protein, MesD (Accession number: Q10418.1). Next to this gene, the *mesE* gene, coding a 458 AA long protein, showed 66.81 % identity to mesentericin Y105 secretion protein, MesE (Accession number: Q10419.1).

Upstream to the mesentericin B105 gene, there were two more genes, encoding bacteriocin class IIc type proteins. The 48 AA protein showed 97.96 % identity to lactococcin G-beta/enterocin 1071B family bacteriocin (Accession number: WP_010015613.1), while the 47 AA protein showed 95.74 % identity to lactococcin G-alpha/enterocin 1071A family bacteriocin (Accession number: WP_224172900.1).

#### Identification of Genes With Potential Relevance for Probiotics

**Fig. 3:**
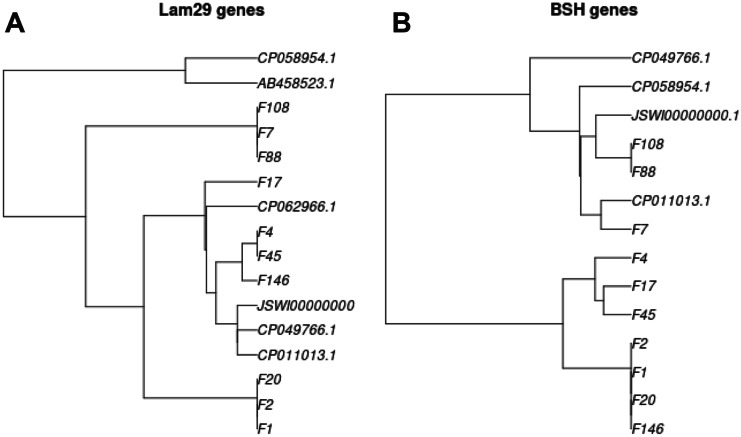
Phylogenetic relationship of *lam29* genes (**A**) and *bsh* genes (**B**) found in *Limosilactobacillus mucosae* strains. Sequences used for comparison with sequences of our strains were obtained from NCBI database and marked with their accession numbers. AB458523.1 refers to ABC2745 gene in *L. mucosae* OLL2745, CP062966.1 refers to *L. mucosae* LM011 in part **A**, and CP011013.1, CP058954.1, CP049766.1 and JSWI00000000.1 refer in both part **A** and **B** to *L. mucosae* strains, such as LM1, A1, L1 and DPC6426, respectively

Gastrointestinal retention of probiotic bacteria is highly dependent on the interaction between bacterial cell surface proteins and the mucus layer of the host gut. In the case of *L. mucosae,* a 29-kDa protein (Lam29), a cysteine-binding protein of an ABC transporter, has been identified previously and its mucin- and epithelial cell-adhesion capability was proved. We have intended to see whether the Lam29 protein gene exist in our strains. The corresponding coding region has been identified in each *L. mucosae* draft genome sequence, and they were compared to sequences of other *L. mucosae* strains found in the NCBI database. Relationships of the alleles are shown in a phylogenetic tree (Fig. 3A). Clustering of F1, F2 and F20 as well as F7, F88 and F108 strains based on relatedness of the *lam29* genes resemble their clustering based on the entire genomes, while clustering of F146 together with F4 and F45 and the position of F17 shows a significant alteration in this context.

It has been shown experimentally that all 47 *L. mucosae* strains expressed reasonable BSH activity, while none out of the 9 *L. suionicum* strains did. We have intended to trace this phenomenon in the obtained sequences as well. No *bsh* gene was found in the *L. suionicum* sequences, while one *bsh* gene was identified in each *L. mucosae* genome, and their relationship is presented in a phylogenetic tree (Fig.3B). Clustering of F1, F2, F20 and F146 as well as F4, F17 and F45 strains based on relatedness of the *bsh* genes is similar to their clustering based on the entire genome sequences. F88 and F108 also clustered together as it could be expected but F7 did not join to this group, it rather showed closer relationship with *L. mucosae* LM1.

## Discussion

Probiotics development has become a promising field of biological investments in the last decade. Despite the fact that large number of probiotic strains have been isolated, characterized and published so far and many of them are commercially available worldwide, discovery of new strains with promising probiotic properties is always desirable, as probiotic features are strain-specific and possess different beneficial properties and effects [3, 57]. In our study we report our efforts to isolate lactic acid bacteria from free-living wild boars to select strains with probiotic values for swine industry.

To establish a primary strain collection, colonies were isolated from gut content at four locations of the gastrointestinal tracts (ileum, colon, caecum and rectum) of five wild boars. The use of six different media in three incubation conditions (aerobic, semi-anaerobic and anaerobic) ensured that a broad spectrum of bacteria was isolated.

For screening the isolates, a PCR-based approach was an efficient way to narrow down the strain collection by using a primer pair, specific for four genera (*Lactobacillus*, *Pediococcus*, *Leuconostoc*, and *Weissella*) [18]. The narrowed collection including the genus *Lactobacillus*, the one that has been found to be safe for consumption (having “Generally Regarded as Safe” (GRAS) status) [6]. PCR positive isolates were further classified by culture-based methods. This step led to further selection of the isolates, since only well-culturable strains are suitable for probiotics development, so strains not meeting this criterium were omitted from further study.

Based on the above criteria, 166 strains were selected for further screening. Since having antimicrobial activity is one of the main requirements for probiotics, the isolates were evaluated for their antibacterial activity against four tester strains and according to their antimicrobial activity pattern, 56 strains were selected for further analysis.

Taxonomic identification at species level, based on 16S rDNA sequence determination and comparison to sequences in reference databases, was essential for further characterization of potential probiotic isolates. It is worth mentioning that a major reclassification of lactobacilli species was performed recently [34], renaming the former species *Lactobacillus mucosae* to *Limosilactobacillus mucosae*. *Leuconostoc suionicum* is a young species, it was first established as a subspecies within the *L. mesenteroides* species [58] and later became a self-standing species [35]. Out of our 166 strains we have identified so far, 61 *L. mucosae* and 11 *L. suionicum* strains (Supplementary Table 1). There is currently no explanation why only these two species were identified from our samples instead of isolating more species, particularly from lactobacilli. However, it remains possible that other species are present among the remaining 94 isolates.

*L. mucosae* was discovered by Roos et al. [59] as a new *Lactobacillus* species with in vitro mucus-binding ability, isolated from pig small intestine. Since then, *L. mucosae* strains were isolated from human source, such as feces [60], ileal epithelium [61], vaginal tract [62] and from bovine intestine and stool [63], as well as from milk of goat [64], sheep [65] and donkey [66]. Recently, the isolation and comparative genome analyses of 93 of *L. mucosae* strains from different niches have been reported [55]. Taxonomic position of the species within the genus *Limosilactobacillus* has been confirmed recently [67]. In parallel to isolation of new *L. mucosae* strains of different origins, the evaluation of the probiotic potential of the species was also performed, confirming that *L. mucosae* strains can be selected as probiotics since they possess a broad spectrum of biological functional attributes [66]. *L. mucosae* strain LM1 has been studied extensively in vitro [59, 68–73] and its probiotic potential was also investigated in vivo [74].

*L. mucosae* strains have been shown to inhibit a number of Gram (+) and Gram (-) pathogens [71], such as *Escherichia coli*, *Salmonella enterica* [69] and *Staphylococcus aureus* [75]. Our results are consistent with these findings, 56 out of the 61 identified *L. mucosae* strains exerted antimicrobial activity against *Escherichia coli*, *Salmonella enterica* and *Staphylococcus aureus*, while 38 of them had inhibited the growth of *Streptococcus thermophilus* (Supplementary Table 1 and Table 1).

Regarding *Leuconostoc suionicum*, the first complete genome sequence was published in 2017 (*L. suionicum* DSM 20241 [76]). Currently, 2 complete and 8 draft genome sequences are available in the NCBI database, and recent reclassification within the *Leuconostoc* genus confirmed the taxonomic status of the species [77]. A high degree of similarity between the genome sequences of *L. suionicum* and *Leuconostoc mesenteroides* was detected, which is not surprising since the previous *L. suionicum* strains belonged to a subspecies of *Leuconostoc mesenteroides* [35]. Studies addressing probiotic assessment of microorganisms in the genus *Leuconostoc*, in particular *L. mesenteroides* species, are very diverse in the literature but no detailed probiotic study has been performed on strains of *L. suionicum* species. It was shown that all 20 *Leuconostoc* strains, including the 11 *L. suionicum* ones exerted antimicrobial activity against *Escherichia coli*, *Salmonella enterica* and *Staphylococcus aureus* but not against *Streptococcus thermophilus* (Supplementary Table 1 and Table 1)*.*

It is very important that probiotic strains do not cause any harm to the host organism, so their safety of use should be monitored in this respect as well. According to safety regulations and standards [1], – “Strains of micro-organisms carrying an acquired resistance to antimicrobial(s) shall not be used as feed additives, unless it can be demonstrated that resistance is a result of chromosomal mutation(s) and it is not transferable.” –, determination of the antibiotic susceptibility profile of potential strains should be performed. The aim is to exclude the presence of any transmissible, acquired antibiotic resistance gene, but the presence of non-transmissible, intrinsic antibiotic resistance genes is acceptable or can be even beneficial. Seven antibiotics (erythromycin, kanamycin, tetracycline, trimethoprim, vancomycin, oxacillin and azithromycin) were used to determine the antibiotic resistance/susceptibility patterns of the selected strains by disc diffusion method.

In general, most *Lactobacillus* species are intrinsically resistant to kanamycin, vancomycin and trimethoprim, while sensitive to tetracycline, erythromycin and oxacillin [78, 79]. However, acquired determinants encoding resistance for tetracycline and erythromycin were detected in some *Lactobacillus* species [79]. It is difficult to say how reliably the vast amount of data and analyses accumulated for the genus *Lactobacillus* can be applied to the genus *Limosilactobacillus*. Data on the susceptibility of *L. mucosae* strains are sparse, but two strains reported were resistant to vancomycin and sensitive to tetracycline and erythromycin [66]. As expected, all 47 *L. mucosae* strains tested were resistant to vancomycin and kanamycin but surprisingly 5 strains were sensitive to trimethoprim. All 47 strains were sensitive to erythromycin. Although none of the strains were resistant to tetracycline, oxacillin and azithromycin, 18, 12 and 10 strains had intermediate phenotype, respectively (Fig. 1). If all strains with intermediate antibiotic resistance are excluded, 21 of the 47 *L. mucosae* strains need to be excluded from further probiotic development.

*Leuconostoc* species have intrinsic resistance to vancomycin and trimethoprim, while atypical resistance to kanamycin, tetracycline and erythromycin has been observed [80]. All 9 *L. suionicum* strains tested were resistant to vancomycin, trimethoprim and kanamycin (Fig. 1), and all strains were sensitive to erythromycin. While vancomycin and trimethoprim resistance are acceptable, the presence of kanamycin resistance raises concerns. It will be necessary to prove their non-transmissible nature if the strains are selected for probiotics development. Intermediate phenotypes were also detected: 1 strain to tetracycline and 2-2 strains showed intermediate resistance to oxacillin and azithromycin respectively (Fig. 1). These results suggest that a total of 3 out of 9 *L. suionicum* strains should be considered for exclusion.

As a molecular approach we have used specific primer pairs to reveal the presence of *blaZ* gene for ampicillin, *tet*(K) for tetracycline, *sat4* for streptothricin, *mph*(C) and *msrA* for macrolides, *mecA* for meticillin and oxacillin, *dfr*(A) for trimethoprim, *aph*(3)-*III* for kanamycin resistance by PCR methods [28]. None of these genes were present in our strains investigated. Since both the disk diffusion antibiotic susceptibility and PCR tests have serious limitation, whole genome sequence could be a good way to assess the safety of the strains. Draft whole genome sequences of ten *L. mucosae* and three *L. suionicum* strains were determined. Bioinformatic analysis ruled out the presence of any known acquired antibiotic resistance gene within these genomes. Based on this result, there is no reason to exclude any of these candidate strains due to presence of prohibited transmissible antibiotic resistance.

Taxonomical identity of the strains was confirmed in three independent ways. Primarily, 16S rDNA sequencing gave convincing results for species-level identification. Using draft genome sequences, K-mer analyses identified the best matching sequences in the database and thus, in addition to species determination, the closest relatives were identified at strain level (Table 3). Finally, analysis of the average nucleotide identity (ANI) of the genomes of our own and some related strains in the NCBI database confirmed the previous results, and phylogenetic trees showed the relationship between the strains investigated (Fig. 2A and B).

Phylogenetic relationship of the strains, as shown by both, the pairwise values of the similarity matrix of the ANI analyses (Supplementary Table 4) and phylogenetic trees (Fig. 2A and B), indicate that our isolation and screening protocol worked well and allowed the identification of distinct strains for both species. *L. mucosae* strain F7, F88 and F108 are closely related to the type strain *L. mucosae* LM1 and some other known strains in the database, while the other 7 strains form a separate subclade (Table 3 and Fig. 2A). The similarity between *L. suionicum* strains F150 and F156 is 99.9999 % based on FastANI analysis, indicating that they could be identical. Although it would not be a surprising result, since they derived from the same sample, but their behavior was different in both, the antimicrobial activity test (Table 1) and antibiotic susceptibility profile (Fig. 1), so they are still considered to be different strains.

Bacteriocin production of some LAB strains may ensure their stable persistence in the microbial population as they may have competitive advantage over other microorganisms. The presence of enterolysin A genes was reported in several *L. mucosae* strains by sequence analyses [55]. However, there was no known bacteriocin gene detected either in our *L. mucosae* strains, or in the closest relative *L. mucosae* LM1, although some of the strains, such as F1, F2, F4 and F7, exhibited strong antimicrobial activity against several different microorganisms.

The sequences of the two very closely related *L. suionicum* strains (F150 and F156) contained such genetic elements that are likely to be involved in antibacterial function, whereas we could not identify bacteriocin related genes in the closely related F151. From the antibacterial factors identified in *Leuconostoc* strains F150 and F156, mesentericin B105 could theoretically be functional, as it has been reported that the MesDE secretion machinery is capable of transporting and maturing different pre-bacteriocins, mesentericin Y105 and B105 [81]. Since the *mesDE* genes in strains F150 and F156 showed significant similarity to the *mesDE* genes of the mesentericin Y105 system, and it has been previously shown that the mesentericin Y105 system is cross-compatible with B105 system [81, 82], and as a consequence, functional mesentericin B105 may be present in F150 and F156 strains. In addition to *mesDE* genes, the mesentericin operon in different *Leuconostoc* strains also contained some additional genes [83], however, we have not identified similar genes in the region.

It is well known that there are multifunctional proteins, sometimes called moonlighting proteins, which perform two or more functions in addition to their primary (originally identified) function [84]. In lactobacilli, the following proteins have been reported to act as mucin adhesion factors: elongation factor Tu (EF-Tu), glyceraldehyde 3-phosphate dehydrogenase (GAPDH), chaperonin GroEL, and ATP-binding cassette (ABC) transporter [85]. The presence of the mucus-binding protein (MUB) leaded to the discovery of the new species, *Lactobacillus mucosae* [59] and mucin-adhesion ability of *L. mucosae* LM1 was tested *in vitro* [69]. The elongation factor Tu, glyceraldehyde 3-phosphate dehydrogenase, and the phosphocarrier protein HPr were upregulated in *L. mucosae* LM1 when co-cultured with intestinal porcine epithelial cells [72]. In *L. mucosae* a 29-kDa protein (Lam29), an ABC transporter component protein has been identified and its mucin- and epithelial cell-adhesion capability was proved [48, 49]. The presence of an orthologue gene of the ABC transporter protein has also been demonstrated in the *L. mucosae* LM1 genome [68].

It is evident that mucin binding ability of the strains investigated needs to be addressed experimentally, since even more than one adhesion factor may be involved, and their combined effect should be determined. However, the Lam29 protein appears to be a *L. mucosae*-specific mucin adhesion factor, and the presence of the *lam29* gene in the sequenced strains raises the possibility that the strains possess one of the desirable properties of probiotics, the ability to bind mucins. The likelihood of the presence of a functional *lam29* gene in our strains investigated is further supported by the fact that the proven mucin-binding *L. mucosae* LM1 was their closest relative in the entire database (Table 3), the relationship of this gene family is presented in Fig. 3A.

Gut microbial enzymes contribute significantly to bile acid metabolism through deconjugation and dehydroxylation reactions to generate unconjugated and secondary bile acids. These microbial enzymes (which include bile salt hydrolase (BSH)) are essential for bile acid homeostasis in the host and represent a vital contribution of the gut microbiome to host health [86].

Conjugated bile acids are toxic to bacteria, particularly at low pH, and can affect the bacteria growth in different regions of the GI tract [87]. BSH can confer a protective effect to certain bacteria through bile acid deconjugation and can be an advantage during bacterial colonization, thus BSH activity can be associated with a higher survival of intestinal transit by rendering them more tolerant to bile salts. Therefore, it has become a selection criterium for probiotics [88–91]. However, it is yet not completely clear whether BSH activity is a desirable property [92], as high levels of certain secondary bile acids have proinflammatory effect [93] and can promote the development of infectious, inflammatory or malignant diseases [94, 95]. On the other hand, abundance of the *bsh* gene was significantly reduced in patients with inflammatory bowel disease (IBD) and type-2 diabetes [96].

BSH enzymes are present in various microbial species in most phyla [89]. It was shown that BSH-positive LAB commonly exists in the GIT and the BSH activity of LAB was correlated with their natural habitat [97]. BSH-encoding lactobacilli have primarily a vertebrate-adapted lifestyle as opposed to environment- or plant-associated lactobacilli [95].

The number of *bsh* alleles can vary, with up to 4 different alleles occurring in certain isolates of different *Lactobacillus* species [98–103], but misclassification of *bsh* genes in databases has also occurred [98, 103]. It was shown by *in silico* analysis of 8 *Lactobacillus mucosae* genomes that all of them contained only one *bsh* gene [98]. More than 300 LAB strains belonging to several genera have been investigated and BSH activity was found in the majority of *Lactobacillus* strains but was absent in *L. mesenteroides* strains [97]. Our results coincide with these findings, since BSH activity was found in all the 47 *L. mucosae* strains investigated (Table 2) and identified one *bsh* gene in each of the 10 sequenced genomes (Fig. 3B). Surprisingly, no *bsh* gene was found in the genome sequence of *L. mucosae* LM011 (Accession number: CP062966.1). On the other hand, our 9 *L. suionicum* strains did not show any BSH activity (Table 2), and three sequenced genomes did not contain any *bsh* gene.

For probiotics development, isolation of candidate bacteria, assessment of their probiotic potential and safety attributes in vitro are only the beginning steps. In this context, a wide range of in vitro attributes should be investigated, preferably using a combination of classical microbiological, molecular biological and genomic methods. In this way, the range of potential probiotic strains can be narrowed down considerably, as we have done in our current work. However, even the most careful in vitro experiments cannot provide a conclusive answer as to which strains are suitable for use as probiotics. As a consequence, potential probiotic strains can and should be tested in targeted animal experiments.

In our study, from the rich primary collection of isolates from different gut contents of wild boars, 56 strains were selected for detailed analyses. The majority of the strains possessed antibacterial activity against four different common bacteria, including pathogenic ones, and were highly tolerant to stress factors, characteristic for gut environment, such as low pH and high bile concentration. None of the strains had hemolytic activity, and there was no sign of having any transmissible antibiotic resistance genes as well. It is still not clear whether high BSH activity will be a desirable attribute for the final probiotic development, but by having the full spectrum (strong, medium and weak activity in *L. mucosae* strains and no activity in *L. suionicum* strains) available, it gives us the opportunity to select strains with the desired characteristics. Further assessment in the course of in vivo feeding experiments is necessary to prove the usefulness of probiotic strains in pig farming.

### Supplementary Information

Below is the link to the electronic supplementary material.

Supplementary file1 (ZIP 412 KB)
