# Supplementary material for: Isolation and Characterization of Lactic Acid Bacteria With Probiotic Attributes From Different Parts of the Gastrointestinal Tract of Free-living Wild Boars in Hungary
Source: Probiotics Antimicrob Proteins. 2023 Jun 23;16(4):1221–39. doi: 10.1007/s12602-023-10113-2 (PMC11322276; doi:10.1007/s12602-023-10113-2)
Supplement: Supplementary file 1 — Supplementary file1 (ZIP 412 KB) [file 12602_2023_10113_MOESM1_ESM.zip › Kereszteny_et_al_Supplementary/Kereszteny_et_al_Supplementary Table 1.pdf]

Journal: Probiotics and Antimicrobial Proteins. Title: Isolation and characterization of lactic acid bacteria with probiotic attributes from different parts of the gastrointestinal tract of free-living wild boars in Hungary **Authors:** Tibor Keresztény, Balázs Libisch, Stephanya Corral Orbe, Tibor Nagy, Zoltán Kerényi, Róbert Kocsis, Katalin Posta, Péter P. Papp and Ferenc Olasz. **Corresponding author**: Ferenc Olasz Agribiotechnology and Precision Breeding for Food Security National Laboratory, Institute of Genetics and Biotechnology, Hungarian University of Agriculture and Life Sciences (MATE), 2100 Gödöllő, Hungary; olasz.ferenc.gyorgy@uni-mate.hu

Supplementary Table 1. **Grouping of 166 LAB isolates based on pattern of their antimicrobial activity using four tester strains.** Groups are separated by lines, activity pattern is shown once in every group for clarity.

**Isolate**		**Isolation**[a]	**PCR screening**[b]		**Taxonomic identification**[c]		**Antimicrobial activity**[d] **against**			
ID	Origin[e]	Plate	Lac1F/ Lac2GCR	Leucgrp fw/rev	Genus	Species	*Escherichia coli*	*Salmonella enterica*	*Staphylococcus aureus*	*Streptococcus thermophylus*
F1	caecum	MRSCC	+	-	*Limosilactobacillus*	*mucosae*	+++	+++	+++	+++
F2	ileum	MRSCC	+	-	*Limosilactobacillus*	*mucosae*				
F3	ileum	MRSCC	+	-	*Limosilactobacillus*	*mucosae*				
F4	ileum	MRSCC	+	-	*Limosilactobacillus*	*mucosae*	+++	++	+++	+++
F5	ileum	MRSCC	+	-	*LPLW*					
F6	ileum	MRSCC	+	-	*Limosilactobacillus*	*mucosae*				
F7	rectum	M17	+	-	*Limosilactobacillus*	*mucosae*				
F8	caecum	MRSCC	+	-	*Limosilactobacillus*	*mucosae*				
F9	caecum	MRSCC	+	-	*Limosilactobacillus*	*mucosae*				
F10	caecum	MRSCC	+	-	*Limosilactobacillus*	*mucosae*	+++	+++	++	+++
F11	caecum	MRSCC	+	-	*Limosilactobacillus*	*mucosae*				
F12	caecum	MRSCC	+	-	*Limosilactobacillus*	*mucosae*				
F13	ileum	MRSCC	+	-	*Limosilactobacillus*	*mucosae*				
F14	ileum	MRSCC	+	-	*Limosilactobacillus*	*mucosae*				
F15	ileum	MRSCC	+	-	*Limosilactobacillus*	*mucosae*	+++	+	+++	+++
F16	ileum	MRSCC	+	-	*Limosilactobacillus*	*mucosae*	+++	+++	+	+++
F17	caecum	MRSCC	+	-	*Limosilactobacillus*	*mucosae*	+++	+++	+++	-

F18	caecum	MRSCC	+	-	*Limosilactobacillus*	*mucosae*				
F19	ileum	MRSCC	+	-	*LPLW*		+++	+++	++	++
F20	ileum	MRSCC	+	-	*Limosilactobacillus*	*mucosae*				
F21	ileum	MRSCC	+	-	*LPLW*					
F22	caecum	MRSCC	+	-	*LPLW*					
F23	caecum	MRSCC	+	-	*Limosilactobacillus*	*mucosae*				
F24	ileum	MRSCC	+	-	*Limosilactobacillus*	*mucosae*	+++	++	++	+++
F25	ileum	MRSCC	+	-	*LPLW*					
F26	ileum	MRSCC	+	-	*LPLW*					
F27	ileum	MRSCC	+	-	*LPLW*					
F28	ileum	MRSCC	+	-	*LPLW*		+++	+++	++	+
F29	ileum	MRSCC	+	-	*Limosilactobacillus*	*mucosae*	+++	++	+++	+
F30	ileum	MRSCC	+	-	*LPLW*					
F31	ileum	MRSCC	+	-	*Limosilactobacillus*	*mucosae*	+++	+	+++	++
F32	ileum	MRSCC	+	-	*LPLW*					
F33	colon	MRSCC	+	-	*Limosilactobacillus*	*mucosae*				
F34	colon	MRSCC	+	-	*LPLW*		+++	++	+	+++
F35	caecum	MRSCC	+	-	*Limosilactobacillus*	*mucosae*				
F36	colon	MRSCC	+	-	*Limosilactobacillus*	*mucosae*	+++	+	+	+++
F37	colon	MRSCC	+	-	*LPLW*					
F38	colon	MRSCC	+	-	*LPLW*					
F39	colon	MRSCC	+	-	*LPLW*					
F40	colon	MRSCC	+	-	*LPLW*					
F41	colon	MRSCC	+	-	*LPLW*					
F42	colon	MRSCC	+	-	*LPLW*					
F43	colon	MRSCC	+	-	*LPLW*					
F44	colon	MRSCC	+	-	*LPLW*					
F45	colon	MRSCC	+	-	*Limosilactobacillus*	*mucosae*	+++	+++	++	-
F46	caecum	MRSCC	+	-	*Limosilactobacillus*	*mucosae*				
F47	ileum	MRSCC	+	-	*Limosilactobacillus*	*mucosae*				
F48	ileum	MRSCC	+	-	*Limosilactobacillus*	*mucosae*				
F49	ileum	MRSCC	+	-	*Limosilactobacillus*	*mucosae*				
F50	ileum	MRSCC	+	-	*LPLW*					
F51	ileum	MRSCC	+	-	*LPLW*					

F52	ileum	MRSCC	+	-	*Limosilactobacillus*	*mucosae*				
F53	ileum	MRSCC	+	-	*LPLW*					
F54	ileum	MRSCC	+	-	*LPLW*					
F55	ileum	MRSCC	+	-	*LPLW*		+++	++	+++	-
F56	ileum	MRSCC	+	-	*LPLW*					
F57	ileum	MRSCC	+	-	*LPLW*					
F58	ileum	MRSCC	+	-	*LPLW*					
F59	ileum	MRSCC	+	-	*LPLW*					
F60	ileum	MRSCC	+	-	*LPLW*					
F61	ileum	MRSCC	+	-	*Limosilactobacillus*	*mucosae*	+++	+	+++	-
F62	ileum	MRSCC	+	-	*Limosilactobacillus*	*mucosae*				
F63	caecum	MRSCC	+	-	*LPLW*					
F64	ileum	MRSCC	+	-	*Limosilactobacillus*	*mucosae*	+++	++	++	++
F65	ileum	MRSCC	+	-	*Limosilactobacillus*	*mucosae*				
F66	caecum	MRSCC	+	-	*Limosilactobacillus*	*mucosae*	++	++	++	+++
F67	caecum	MRSCC	+	-	*LPLW*		+++	++	++	+
F68	ileum	MRSCC	+	-	*Limosilactobacillus*	*mucosae*				
F69	ileum	MRSCC	+	-	*Limosilactobacillus*	*mucosae*	+++	+	++	+
F70	caecum	MRSCC	+	-	*LPLW*		+++	+	+	++
F71	colon	MRSCC	+	-	*Limosilactobacillus*	*mucosae*				
F72	colon	MRSCC	+	-	*LPLW*					
F73	colon	MRSCC	+	-	*LPLW*		++	+	+	+++
F74	colon	MRSCC	+	-	*Limosilactobacillus*	*mucosae*				
F75	colon	MRSCC	+	-	*LPLW*					
F76	colon	MRSCC	+	-	*LPLW*					
F77	colon	MRSCC	+	-	*LPLW*		+	+	+	+++
F78	colon	MRSCC	+	-	*LPLW*					
F79	rectum	MRSCC	+	-	*Limosilactobacillus*	*mucosae*				
F80	colon	MRSCC	+	-	*LPLW*					
F81	colon	MRSCC	+	-	*LPLW*					
F82	colon	MRSCC	+	-	*LPLW*					
F83	colon	MRSCC	+	-	*LPLW*					
F84	colon	MRSCC	+	-	*Limosilactobacillus*	*mucosae*				
F85		MRSCC	+	-	*LPLW*					

F86	colon	MRSCC	+	-	*LPLW*					
F87	colon	MRSCC	+	-	*LPLW*					
F88	colon	MRSCC	+	-	*Limosilactobacillus*	*mucosae*				
F89	colon	MRSCC	+	-	*LPLW*					
F90	colon	MRSCC	+	-	*LPLW*					
F91	caecum	MRSCC	+	-	*LPLW*					
F92	caecum	MRSCC	+	-	*LPLW*					
F93	caecum	MRSCC	+	-	*LPLW*		+++	++	++	-
F94	caecum	MRSCC	+	-	*LPLW*					
F95	caecum	MRSCC	+	-	*LPLW*					
F96	caecum	MRSCC	+	-	*LPLW*					
F97	caecum	MRSCC	+	-	*LPLW*					
F98	caecum	MRSCC	+	-	*Limosilactobacillus*	*mucosae*				
F99	ileum	MRSCC	+	-	*LPLW*					
F100	ileum	MRSCC	+	-	*LPLW*					
F101	ileum	MRSCC	+	-	*LPLW*					
F102	ileum	MRSCC	+	-	*Limosilactobacillus*	*mucosae*				
F103	ileum	MRSCC	+	-	*LPLW*					
F104	ileum	MRSCC	+	-	*LPLW*					
F105	ileum	MRSCC	+	-	*Limosilactobacillus*	*mucosae*				
F106	ileum	MRSCC	+	-	*LPLW*					
F107	ileum	MRSCC	+	-	*Limosilactobacillus*	*mucosae*				
	rectum									
F108	ileum	MRSCC	+	-	*Limosilactobacillus*	*mucosae*	++	+++	++	+
F109	colon	MRSCC	+	-	*LPLW*		++	+++	++	-
F110	colon	MRSCC	+	-	*LPLW*		+++	++	+	-
F111	colon	MRSCC	+	-	*LPLW*					
F112	ileum	MRSCC	+	-	*LPLW*		+++	+	++	-
F113	ileum	MRSCC	+	-	*Limosilactobacillus*	*mucosae*	++	+	+++	-
F114	colon	MRSCC	+	-	*LPLW*		+++	+	+	-
F115	caecum	MRSCC	+	-	*LPLW*					
F116	caecum	MRSCC	+	-	*Limosilactobacillus*	*mucosae*	++	++	++	++
F117	caecum	MRSCC	+	-	*LPLW*		++	++	++	+
F118	rectum	MRSCC	+	-	*LPLW*					

F119	rectum	MRSCC	+	-	*LPLW*		++	+	+	++
F120	colon	MRSCC	+	-	*Limosilactobacillus*	*mucosae*				
F121	colon	MRSCC	+	-	*LPLW*		++	+	+	+
F122	colon	MRSCC	+	-	*Limosilactobacillus*	*mucosae*	+	+	++	+
F123	colon	MRSCC	+	-	*LPLW*		+	+	+	++
F124	colon	MRSCC	+	-	*LPLW*					
F125	colon	MRSCC	+	-	*LPLW*					
F126	caecum	MRSCC	+	-	*Limosilactobacillus*	*mucosae*				
F127	caecum	MRSCC	+	-	*LPLW*					
F128	caecum	MRSCC	+	-	*LPLW*		++	++	++	-
F129	caecum	MRSCC	+	-	*LPLW*					
F130	caecum	MRSCC	+	-	*LPLW*					
F131	caecum	MRSCC	+	-	*LPLW*					
F132	ileum	MRSCC	+	-	*Limosilactobacillus*	*mucosae*				
F133	colon	MRSCC	+	-	*Limosilactobacillus*	*mucosae*				
F134	colon	MRSCC	+	-	*LPLW*		++	++	+	-
F135	ileum	MRSCC	+	-	*Limosilactobacillus*	*mucosae*				
F136	colon	MRSCC	+	-	*LPLW*		++	+	+	-
F137	ileum	MRSCC	+	-	*Limosilactobacillus*	*mucosae*	++	++	-	-
F138	ileum	MRSCC	+	-	*Limosilactobacillus*	*mucosae*	++	-	-	-
F139	rectum	MRSCC	+	-	*Limosilactobacillus*	*mucosae*	+	+	+	+
F140	caecum	MRSCC	+	-	*LPLW*		+	-	+	-
F141	caecum	MRSCC	+	-	*LPLW*		+	-	-	+
F142	colon	MRSCC	+	-	*LPLW*					
F143	caecum	MRSCC	+	-	*Limosilactobacillus*	*mucosae*	+	-	-	-
F144	caecum	MRSCC	+	-	*Limosilactobacillus*	*mucosae*				
F145	colon	MRSCC	+	-	*LPLW*		-	-	-	-
F146	caecum	MRSCC	+	-	*Limosilactobacillus*	*mucosae*				
F147	caecum	M17	+	+	*Leuconostoc*	*suionicum*				
F148	caecum	MRSCC	+	+	*Leuconostoc*	*suionicum*	+++	++	++	-
F149	caecum	ELLIKER	+	+	*Leuconostoc*	*suionicum*				
F150	caecum	MRSCC	+	+	*Leuconostoc*	*suionicum*				
F151	rectum	MRSCC	+	+	*Leuconostoc*	*suionicum*	+++	++	+	-

F152	caecum	ELLIKER	+	+	*Leuconostoc*	*suionicum*				
F153	caecum	M17	+	+	*Leuconostoc*					
F154	caecum	M17	+	+	*Leuconostoc*					
F155	caecum	M17	+	+	*Leuconostoc*					
F156	caecum	M17	+	+	*Leuconostoc*	*suionicum*				
F157	caecum	ELLIKER	+	+	*Leuconostoc*					
F158	rectum	MRS	+	+	*Leuconostoc*					
F159	rectum	MRS	+	+	*Leuconostoc*		++	+	+	-
F160	rectum	MRS	+	+	*Leuconostoc*					
F161	rectum	MRS	+	+	*Leuconostoc*					
F162	rectum	ELLIKER	+	+	*Leuconostoc*	*suionicum*				
F163	rectum	MRSCC	+	+	*Leuconostoc*	*suionicum*				
F164	rectum	MRSCC	+	+	*Leuconostoc*					
F165	rectum	MRSCC	+	+	*Leuconostoc*					
F166	rectum	MRSCC	+	+	*Leuconostoc*	*suionicum*				

a – "Plates" reflects to the original agar medium used to obtain isolates at the first time, growth condition was anaerobic for all strains except for F7 which was semi-anaerobic

b – "+" marks positive detection of expected specific PCR fragment; "-" means: no specific fragment amplified

c – "*LPLW*" indicates that the given isolate can belong to any of the *Lactobacillus, Pediococcus, Leuconostoc* or *Weissella* genera, based on the positive results in PCR with Lac1F and Lac2GCR primer pair. *Leuconostoc* genus is specified if PCR with Leucgrp fw and Leucgrp rev primer pairs gave also positive result. Taxonomic identification at species level is based on 16S rDNA sequencing.

d – Activity is rated according to the size of clear inhibition zone around the wells, diameter rating is: –: 6 mm; +: >6-9 mm; ++: >9-15 mm and +++: >15 mm

e – gut region of sample origin
