# Supplementary material for: Isolation and Characterization of Lactic Acid Bacteria With Probiotic Attributes From Different Parts of the Gastrointestinal Tract of Free-living Wild Boars in Hungary
Source: Probiotics Antimicrob Proteins. 2023 Jun 23;16(4):1221–39. doi: 10.1007/s12602-023-10113-2 (PMC11322276; doi:10.1007/s12602-023-10113-2)
Supplement: Supplementary file 1 — Supplementary file1 (ZIP 412 KB) [file 12602_2023_10113_MOESM1_ESM.zip › Kereszteny_et_al_Supplementary/Kereszteny_et_al_Supplementary Table 2.pdf]

Journal: Probiotics and Antimicrobial Proteins. Title: Isolation and characterization of lactic acid bacteria with probiotic attributes from different parts of the gastrointestinal tract of free-living wild boars in Hungary **Authors:** Tibor Keresztény, Balázs Libisch, Stephanya Corral Orbe, Tibor Nagy, Zoltán Kerényi, Róbert Kocsis, Katalin Posta, Péter P. Papp and Ferenc Olasz. **Corresponding author**: Ferenc Olasz Agribiotechnology and Precision Breeding for Food Security National Laboratory, Institute of Genetics and Biotechnology, Hungarian University of Agriculture and Life Sciences (MATE), 2100 Gödöllő, Hungary; olasz.ferenc.gyorgy@uni-mate.hu

Supplementary Table 2. Viable cell counts of isolates after 1 h and 2h incubation at pH 2.5 (log CFU/mL)

Strain ID	Initial concentration (log unit)	pH 2.5			
		1 h (log unit)	change (log unit)	2 h (log unit)	change (log unit)
L. mucosae					
F1	7.04 ± 0.08	6.72 ± 0.04	- 0.32	7.20 ± 0.06	+ 0.16
F2	7.77 ± 0.04	7.60 ± 0.01	- 0.17	7.58 ± 0.06	- 0.19
F4	7.17 ± 0.06	7.87 ± 0.10*	+ 0.70	7.84 ± 0.10*	+ 0.67
F6	7.57 ± 0.06	7.57 ± 0.04	0.00	7.37 ± 0.02	- 0.20
F7	7.61 ± 0.10	7.23 ± 0.08	- 0.38	6.96 ± 0.09	- 0.65
F9	7.69 ± 0.01	7.41 ± 0.01	- 0.28	7.23 ± 0.04	- 0.46
F10	7.17 ± 0.04	7.81 ± 0.05	+ 0.64	5.36 ± 0.18**	- 1.81
F13	6.87 ± 0.05	7.02 ± 0.08	+ 0.15	6.85 ± 0.13	- 0.02
F14	7.22 ± 0.03	7.52 ± 0.20	+ 0.30	7.44 ± 0.24	+ 0.22
F15	7.10 ± 0.07	6.91 ± 0.07	- 0.19	6.95 ± 0.01	- 0.15
F16	7.05 ± 0.09	7.05 ± 0.14	0.00	7.19 ± 0.09	+ 0.14
F17	7.21 ± 0.07	7.19 ± 0.16	- 0.02	7.23 ± 0.07	+ 0.02
F18	7.08 ± 0.04	7.12 ± 0.04	+ 0.04	7.15 ± 0.02	+ 0.07
F20	7.34 ± 0.05	7.25 ± 0.11	- 0.09	7.08 ± 0.12	- 0.26
F23	6.41 ± 0.09	6.81 ± 0.20	+ 0.40	7.28 ± 0.09*	+ 0.87
F24	7.00 ± 0.06	7.05 ± 0.28	+ 0.05	7.19 ± 0.13	+ 0.19
F29	6.93 ± 0.06	7.07 ± 0.13	+ 0.14	7.08 ± 0.02	+ 0.15
F31	7.01 ± 0.05	7.51 ± 0.18	+ 0.50	7.65 ± 0.16	+ 0.64
F35	7.38 ± 0.03	7.59 ± 0.05	+ 0.21	7.64 ± 0.05	+ 0.26
F45	7.30 ± 0.04	7.34 ± 0.24	+ 0.04	7.57 ± 0.05	+ 0.27
F48	6.78 ± 0.14	7.08 ± 0.32	+ 0.30	6.97 ± 0.16	+ 0.19
F49	6.71 ± 0.52	7.75 ± 0.04*	+ 1.04	7.39 ± 0.15*	+ 0.68
F52	6.98 ± 0.04	7.35 ± 0.20	+ 0.37	7.49 ± 0.20	+ 0.51
F61	6.88 ± 0.11	7.27 ± 0.15	+ 0.39	7.48 ± 0.03	+ 0.60
F65	7.16 ± 0.02	6.99 ± 0.14	- 0.17	6.89 ± 0.12	- 0.27
F66	7.27 ± 0.10	7.45 ± 0.07	+ 0.18	7.55 ± 0.04	+ 0.28
F68	6.76 ± 0.16	7.47 ± 0.19*	+ 0.71	7.44 ± 0.18*	+ 0.68
F69	7.22 ± 0.06	7.60 ± 0.29	+ 0.38	7.48 ± 0.18	+ 0.26
F71	7.12 ± 0.30	7.94 ± 0.20	+ 0.82	7.70 ± 0.20	+ 0.58
F79	7.40 ± 0.01	7.56 ± 0.03	+ 0.16	7.52 ± 0.07	+ 0.12

F84	7.60 ± 0.05	7.47 ± 0.05	- 0.13	7.44 ± 0.12	- 0.16
F88	7.29 ± 0.09	6.98 ± 0.02	- 0.31	6.77 ± 0.08	- 0.52
F98	7.19 ± 0.08	7.05 ± 0.25	- 0.14	6.66 ± 0.29	- 0.53
F105	6.90 ± 0.03	3.00 ± 0.25**	- 3.90	3.75 ± 0.08**	- 3.15
F108	7.57 ± 0.10	7.78 ± 0.05	+ 0.21	7.53 ± 0.11	- 0.04
F113	7.23 ± 0.02	7.70 ± 0.25	+ 0.47	7.81 ± 0.18	+ 0.58
F116	6.98 ± 0.09	7.25 ± 0.21	+ 0.27	7.70 ± 0.26*	+ 0.72
F120	6.26 ± 0.28	4.62 ± 0.09**	- 1.64	4.36 ± 0.07**	- 1.90
F122	6.75 ± 0.04	5.92 ± 0.20	- 0.83	5.46 ± 0.03**	- 1.29
F126	6.13 ± 0.04	6.10 ± 0.14	- 0.03	5.69 ± 0.29	- 0.44
F132	5.95 ± 0.39	7.36 ± 0.04*	+ 2.41	7.37 ± 0.04*	+ 1.42
F133	7.34 ± 0.11	7.24 ± 0.19	- 0.10	7.00 ± 0.06	- 0.34
F137	6.78 ± 0.12	6.99 ± 0.11	+ 0.21	7.02 ± 0.10	+ 0.24
F138	7.53 ± 0.17	7.72 ± 0.02	+ 0.19	7.74 ± 0.03	+ 0.21
F139	7.66 ± 0.02	7.53 ± 0.07	- 0.13	7.47 ± 0.06	- 0.19
F144	6.46 ± 0.09	6.33 ± 0.08	- 0.13	6.42 ± 0.04	- 0.04
F146	7.79 ± 0.03	7.76 ± 0.08	- 0.03	7.67 ± 0.07	- 0.13
L. suionicum					
F147	7.34 ± 0.07	4.32 ± 0.10**	- 3.02	0.00 ± 0.00**	- 7.00
F148	7.13 ± 0.04	0.87 ± 0.00**	- 6.26	0.00 ± 0.00**	- 7.13
F150	7.34 ± 0.04	5.20 ± 0.04**	- 2.14	0.00 ± 0.00**	- 7.34
F151	7.54 ± 0.01	5.88 ± 0.04**	- 1.66	0.00 ± 0.00**	- 7.54
F156	7.38 ± 0.02	5.80 ± 0.11**	- 1.58	0.00 ± 0.00**	- 7.38
F158	7.34 ± 0.07	0.00 ± 0.00**	- 7.34	0.00 ± 0.00**	- 7.34
F162	7.54 ± 0.02	0.00 ± 0.00**	- 7.54	0.00 ± 0.00**	- 7.54
F163	7.57 ± 0.01	5.86 ± 0.04**	- 1.71	0.00 ± 0.00**	- 7.57
F166	7.41 ± 0.02	0.00 ± 0.00**	- 7.41	0.00 ± 0.00**	- 7.41

* – viable counts were increased significantly (over 0.7 log unit increase)

** – viable counts were decreased significantly (over 1.0 log unit decrease)
