# Supplementary material for: Isolation and Characterization of Lactic Acid Bacteria With Probiotic Attributes From Different Parts of the Gastrointestinal Tract of Free-living Wild Boars in Hungary
Source: Probiotics Antimicrob Proteins. 2023 Jun 23;16(4):1221–39. doi: 10.1007/s12602-023-10113-2 (PMC11322276; doi:10.1007/s12602-023-10113-2)
Supplement: Supplementary file 1 — Supplementary file1 (ZIP 412 KB) [file 12602_2023_10113_MOESM1_ESM.zip › Kereszteny_et_al_Supplementary/Kereszteny_et_al_Supplementary Table 3.pdf]

Journal: Probiotics and Antimicrobial Proteins. Title: Isolation and characterization of lactic acid bacteria with probiotic attributes from different parts of the gastrointestinal tract of free-living wild boars in Hungary **Authors:** Tibor Keresztény, Balázs Libisch, Stephanya Corral Orbe, Tibor Nagy, Zoltán Kerényi, Róbert Kocsis, Katalin Posta, Péter P. Papp and Ferenc Olasz. **Corresponding author**: Ferenc Olasz Agribiotechnology and Precision Breeding for Food Security National Laboratory, Institute of Genetics and Biotechnology, Hungarian University of Agriculture and Life Sciences (MATE), 2100 Gödöllő, Hungary; olasz.ferenc.gyorgy@uni-mate.hu

Supplementary Table 3. Viable cell counts of isolates after 4 h and 7 h incubation at 0.3 % and 1.0 % bile concentration (log CFU/mL)

Strain ID	Initial concentration (log unit)	0.3 % bile				1.0 % bile			
		4 h (log unit)	4 h change (log unit)	7 h (log unit)	7 h change (log unit)	4 h (log unit)	4 h change (log unit)	7 h (log unit)	7 h change (log unit)
L. mucosae									
F1	7.66±0.05	7.65±0.03	- 0.01	7.56±0.03	- 0.10	6.18±0.07	- 1.48	6.06±0.09	- 1.60
F2	7.80±0.04	7.78±0.06	- 0.02	7.69±0.07	- 0.11	7.43±0.03*	- 0.37	7.41±0.04*	- 0.39
F4	7.68±0.01	7.65±0.02	- 0.03	7.18±0.03	- 0.50	7.24±0.10*	- 0.44	7.24±0.05*	- 0.44
F6	7.57±0.06	7.47±0.02	- 0.10	7.41±0.05	- 0.16	6.54±0.13	- 1.03	6.57±0.01	- 1.00
F7	7.12±0.07	7.11±0.03	- 0.01	7.47±0.04	+ 0.35	6.86±0.04*	- 0.26	6.81±0.08*	- 0.31
F9	7.39±0.03	6.95±0.12	- 0.44	6.91±0.07	- 0.48	6.63±0.04	- 0.76	6.61±0.08	- 0.78
F10	7.77±0.07	7.77±0.01	0.00	7.85±0.05	+ 0.08	7.07±0.04	- 0.70	7.16±0.12	- 0.61
F13	7.24±0.04	7.33±0.04	+ 0.09	6.12±0.10**	- 1.12	6.42±0.05	- 0.82	6.31±0.07	- 0.93
F14	7.32±0.01	7.05±0.09	- 0.27	6.85±0.08	- 0.47	6.76±0.07	- 0.56	6.57±0.05	- 0.75
F15	6.89±0.03	6.60±0.01	- 0.29	7.05±0.08	+ 0.16	3.63±0.47**	- 3.26	3.33±0.47**	- 3.56
F16	7.09±0.04	6.85±0.03	- 0.24	6.86±0.06	- 0.23	4.40±0.14**	- 2.69	3.93±0.45**	- 3.16
F17	6.82±0.24	6.81±0.06	- 0.01	6.83±0.03	+ 0.01	6.40±0.06	- 0.42	6.29±0.11	- 0.53

F18	7.21±0.07	7.40±0.08	+ 0.19	7.38±0.03	+ 0.17	5.11±0.04	- 2.10	5.01±0.09	- 2.20
F20	7.51±0.05	7.77±0.02	+ 0.26	8.00±0.04	+ 0.49	7.51±0.03*	0.00	7.49±0.03*	- 0.02
F23	7.31±0.04	7.20±0.12	- 0.11	6.71±0.06	- 0.60	6.32±0.12	- 0.99	6.29±0.04	- 1.02
F24	6.92±0.16	7.33±0.03	+ 0.41	7.19±0.05	+ 0.27	6.13±0.02	- 0.79	6.27±0.23	- 0.65
F29	6.80±0.02	6.94±0.07	+ 0.14	7.02±0.02	+ 0.22	6.14±0.02	- 0.66	6.02±0.06	- 0.78
F31	7.16±0.04	7.41±0.03	+ 0.25	7.44±0.08	+ 0.28	6.47±0.04	- 0.69	6.53±0.05	- 0.63
F35	7.61±0.04	7.79±0.02	+ 0.18	7.89±0.02	+ 0.28	7.21±0.02*	- 0.40	7.28±0.04*	- 0.33
F45	6.60±0.01	6.49±0.09	- 0.11	5.37±0.05**	- 1.23	6.40±0.06*	- 0.20	6.18±0.01*	- 0.42
F48	7.30±0.02	7.11±0.02	- 0.19	7.08±0.02	- 0.22	6.07±0.03	- 1.23	6.20±0.07	- 1.10
F49	7.26±0.07	7.17±0.14	- 0.09	7.28±0.11	+ 0.02	5.83±0.05	- 1.43	5.84±0.04	- 1.42
F52	6.94±0.13	7.02±0.12	+ 0.08	6.91±0.02	- 0.03	5.57±0.07	- 1.37	5.76±0.06	- 1.18
F61	7.01±0.11	6.80±0.12	- 0.21	6.62±0.07	- 0.39	5.56±0.18	- 1.45	5.34±0.31	- 1.67
F65	7.27±0.05	7.68±0.03	+ 0.41	7.69±0.06	+ 0.42	6.67±0.05	- 0.60	6.64±0.08	- 0.63
F66	7.23±0.02	7.25±0.06	+ 0.02	7.37±0.09	+ 0.14	6.10±0.03	- 1.13	6.08±0.06	- 1.15
F68	6.76±0.14	6.85±0.13	+ 0.09	6.42±0.35	- 0.34	6.14±0.08	- 1.62	6.08±0.18	- 1.68
F69	7.46±0.04	6.51±0.10**	- 0.95	6.32±0.06**	- 1.14	6.41±0.01	- 1.05	6.31±0.04	- 1.15
F71	7.24±0.04	7.04±0.10	- 0.20	6.98±0.06	- 0.26	3.94±0.18**	- 3.30	3.99±0.03**	- 3.25
F79	6.79±0.02	6.97±0.03	+ 0.18	7.02±0.03	+ 0.23	5.86±0.06	- 0.93	5.80±0.07	- 0.99
F84	6.39±0.04	6.32±0.03	- 0.07	6.45±0.06	+ 0.06	6.31±0.01	- 0.08*	6.37±0.10*	- 0.02
F88	5.95±0.25	6.39±0.05	+ 0.44	6.57±0.01	+ 0.62	5.65±0.16	- 0.30*	6.16±0.10*	+ 0.21
F98	7.14±0.04	7.01±0.26	- 0.13	6.99±0.24	- 0.15	6.34±0.01	- 0.80	6.41±0.04	- 0.73
F105	6.19±0.16	5.89±0.01	- 0.30	5.74±0.10	- 0.45	5.98±0.02	- 0.21	5.67±0.09	- 0.52

F108	7.08±0.03	7.71±0.02	+ 0.63	7.76±0.05	+ 0.68	6.50±0.04	- 0.58	6.49±0.02	- 0.59
F113	6.98±0.08	7.40±0.02	+ 0.42	7.58±0.02	+ 0.60	5.93±0.09	- 1.05	6.12±0.12	- 0.86
F116	7.12±0.17	6.95±0.18	- 0.17	7.11±0.04	- 0.01	6.03±0.07	- 1.09	6.20±0.06	- 0.92
F120	7.18±0.09	7.51±0.05	+ 0.33	7.56±0.04	+ 0.38	5.45±0.00	- 1.73	5.36±0.13	- 1.82
F122	7.30±0.06	7.48±0.05	+ 0.18	7.55±0.03	+ 0.25	5.41±0.10	- 1.89	5.27±0.06	- 2.03
F126	7.27±0.06	7.09±0.07	- 0.18	7.15±0.06	- 0.12	5.41±0.05	- 1.86	5.33±0.17	- 1.94
F132	7.28±0.05	7.19±0.08	- 0.09	7.02±0.04	- 0.26	4.46±0.22**	- 2.82	4.66±0.26**	- 2.62
F133	6.01±0.09	6.42±0.05	+ 0.41	6.38±0.13	+ 0.37	5.68±0.04*	- 0.33	5.60±0.05*	- 0.41
F137	7.75±0.02	7.53±0.06	- 0.22	8.22±0.03	+ 0.47	6.15±0.07	- 1.60	6.47±0.20	- 1.28
F138	7.55±0.05	7.68±0.02	+ 0.13	7.75±0.01	+ 0.20	6.20±0.01	- 1.35	6.20±0.01	- 1.35
F139	6.65±0.11	7.00±0.09	+ 0.35	7.48±0.03*	+ 0.83	6.34±0.05	- 0.31	6.15±0.10	- 0.50
F144	7.29±0.01	7.30±0.03	+ 0.01	7.32±0.02	+ 0.03	6.61±0.04	- 0.68	6.66±0.02	- 0.63
F146	7.77±0.06	7.65±0.07	- 0.12	7.73±0.01	- 0.04	6.35±0.03	- 1.42	6.60±0.17	- 1.17
L. suionicum									
F147	7.45±0.05	7.39±0.03	- 0.06	7.21±0.02	- 0.24	6.81±0.03	- 0.64	6.78±0.03	- 0.67
F148	7.06±0.04	6.96±0.03	- 0.10	6.96±0.06	- 0.10	6.39±0.06	- 0.67	6.49±0.03	- 0.57
F150	7.32±0.04	7.35±0.03	+ 0.03	7.28±0.03	- 0.04	7.19±0.02*	- 0.13	7.17±0.01*	- 0.15
F151	7.22±0.07	8.01±0.16*	+ 0.79	8.16±0.03*	+ 0.94	7.33±0.06*	+ 0.11	7.20±0.04*	- 0.02
F156	6.33±0.03	6.35±0.04	+ 0.02	6.24±0.01	- 0.09	6.20±0.02*	- 0.13	6.16±0.02*	- 0.17
F158	6.25±0.04	6.53±0.17	+ 0.28	6.88±0.01	+ 0.63	6.36±0.05*	+ 0.11	6.28±0.02*	+ 0.03
F162	6.21±0.08	6.23±0.04	+ 0.02	6.14±0.04	- 0.07	6.15±0.02	- 0.06	5.40±0.18	- 0.81

F163	6.23±0.03	6.24±0.04	+ 0.01	5.91±0.17	- 0.32	6.17±0.02	- 0.06	5.40±0.53	- 0.83
F166	7.74±0.05	8.21±0.03	+ 0.47	8.35±0.04	+ 0.61	6.48±0.22	- 1.26	6.47±0.03	- 1.27

* – viable counts were increased from initial counts or decrease was less than 0.4 log unit

** – viable counts were decreased significantly (over 1.0 log unit at 0.3 % bile and over 2.5 log units at 1.0 % bile)
