# Supplementary material for: Isolation and Characterization of Lactic Acid Bacteria With Probiotic Attributes From Different Parts of the Gastrointestinal Tract of Free-living Wild Boars in Hungary
Source: Probiotics Antimicrob Proteins. 2023 Jun 23;16(4):1221–39. doi: 10.1007/s12602-023-10113-2 (PMC11322276; doi:10.1007/s12602-023-10113-2)
Supplement: Supplementary file 1 — Supplementary file1 (ZIP 412 KB) [file 12602_2023_10113_MOESM1_ESM.zip › Kereszteny_et_al_Supplementary/Kereszteny_et_al_Supplementary Table 4.pdf]

Journal: Probiotics and Antimicrobial Proteins. Title: Isolation and characterization of lactic acid bacteria with probiotic attributes from different parts of the gastrointestinal tract of free-living wild boars in Hungary **Authors:** Tibor Keresztény, Balázs Libisch, Stephanya Corral Orbe, Tibor Nagy, Zoltán Kerényi, Róbert Kocsis, Katalin Posta, Péter P. Papp and Ferenc Olasz. **Corresponding author**: Ferenc Olasz Agribiotechnology and Precision Breeding for Food Security National Laboratory, Institute of Genetics and Biotechnology, Hungarian University of Agriculture and Life Sciences (MATE), 2100 Gödöllő, Hungary; olasz.ferenc.gyorgy@uni-mate.hu

Supplementary Table 4. **Similarity data matrix generated by FastANI to compare genome sequences of *Limosilactobacillus* (A) and *Leuconostoc* strains.**

A

Limosilactobacillus	LM1	A1	L1	DPC6426	F108	F146	F17	F1	F20	F2	F45	F4	F7	F88	LM011
L.mucosae LM1 (CP011013.1)	×														
L.mucosae A1 (CP058954.1)	97,4508	×													
L.mucosae L1 (CP049766.1)	96,8483	97.0690	×												
L.mucosae DPC6426 (JSWI00000000.1)	97,2605	98.5620	96.9795	×											
L.mucosae F108	97,6152	97.3601	96.8531	97.0854	×										
L.mucosae F1146	88,1573	87.8768	87.4220	87.5787	88.4975	×									
L.mucosae F17	88,1134	87.9326	87.3359	87.7541	88.7953	97.0743	×								
L.mucosae F1	88,0344	87.8608	87.4114	87.5318	88.5448	99.7500	97.1901	×							
L.mucosae F20	88,1497	87.9179	87.5679	87.5620	88.6003	99.7843	97.1288	99.9657	×						
L.mucosae F2	88,7186	88.2546	87.9411	87.9269	89.3448	99.4999	96.8263	99.6401	99.6897	×					
L.mucosae F45	88,0533	87.6431	87.4407	87.5531	88.8145	97.1920	98.4912	97.2529	97.2112	96.8801	×				
L.mucosae F4	88,3058	88.0107	87.7504	87.8584	88.8760	97.0240	98.6952	97.0560	97.0109	96.6449	98.4279	×			

L.mucosae F7	97,4301	97.4968	96.7066	97.4561	98.1069	88.6450	88.9798	88.5512	88.6779	89.2584	88.9103	89.0577	×		
L.mucosae F88	97,5554	97.3414	96.8147	97.0662	99.9901	88.5969	88.8213	88.5716	88.6492	89.4113	88.7788	88.9274	98.0484	×	
L.mucosae LM011 (CP062966.1)	97,7136	97.3708	96.7039	97.2822	97.6874	88.4659	88.1413	88.4795	88.5294	88.7177	88.2817	88.3088	97.5366	97.6942	×

B

Leuconostoc	LT-38	DSM 20241	WiKim33	CBA 7131	SRCM 102735	SRCM 103456	SRCM 103453	SRCM 103460	MTCC 10508	F150	F151	F156
L. suionicum LT-38 (AP017935.1)	×											
L. suionicum DSM 20241 (CP015247.1)	99.9838	×										
L. mesenteroides WiKim33 (CP021491.1)	94.3367	94.3047	×									
L. mesenteroides CBA7131 (CP021966.1)	94.3065	94.2176	99.6411	×								
L. mesenteroides SRCM102735 (CP028255.1)	94.3600	94.2826	99.7714	99.6911	×							
L. mesenteroides SRCM103456 (CP035139.1)	94.2913	94.2264	99.6134	99.4729	99.6866	×						
L. mesenteroides SRCM103453 (CP035271.1)	94.3304	94.3173	99.6619	99.7273	99.6192	99.6205	×					
L. mesenteroides SRCM103460 (CP035746.1)	94.2879	94.3229	99.6217	99.5334	99.5379	99.4967	99.3752	×				
L. suionicum MTCC10508 (CP058345.1)	99.6405	99.5819	94.3168	94.0862	94.3077	94.2316	94.1606	94.2973	×			
L. suionicum F150	97.6021	97.6388	94.3821	94.2512	94.3774	94.2830	94.2416	94.2344	97.7390	×		
L. suionicum F151	97.5575	97.5922	94.5683	94.1487	94.3283	94.5012	94.1873	94.2976	97.7419	98.5941	×	
L. suionicum F156	97.6022	97.6389	94.3821	94.2512	94.3774	94.2830	94.2416	94.2344	97.7391	99.9999	98.5944	×
